# Medium Cut-Off (MCO) Membranes Reduce Inflammation in Chronic Dialysis Patients—A Randomized Controlled Clinical Trial

**DOI:** 10.1371/journal.pone.0169024

**Published:** 2017-01-13

**Authors:** Daniel Zickler, Ralf Schindler, Kevin Willy, Peter Martus, Michael Pawlak, Markus Storr, Michael Hulko, Torsten Boehler, Marcus A. Glomb, Kristin Liehr, Christian Henning, Markus Templin, Bogusz Trojanowicz, Christof Ulrich, Kristin Werner, Roman Fiedler, Matthias Girndt

**Affiliations:** 1 Charité-Universitaetsmedizin Berlin, Campus Virchow Clinic, Department of Nephrology and Intensive Care Medicine, Berlin, Germany; 2 Institute for Clinical Epidemiology and Applied Biometry, University of Tübingen, Tübingen, Germany; 3 NMI Technology Transfer GmbH, Reutlingen, Germany; 4 Department of Research and Development, Gambro Dialysatoren GmbH, Hechingen, Germany; 5 Institute for Chemistry, Food Chemistry, Martin-Luther-University Halle, Halle an der Saale, Germany; 6 Department of Internal Medicine II, Martin-Luther-University Halle, Halle an der Saale, Germany; Medizinische Universitat Graz, AUSTRIA

## Abstract

**Background:**

To increase the removal of middle-sized uremic toxins a new membrane with enhanced permeability and selectivity, called Medium Cut-Off membrane (MCO-Ci) has been developed that at the same time ensures the retention of albumin. Because many middle-sized substances may contribute to micro-inflammation we hypothesized that the use of MCO-Ci influences the inflammatory state in hemodialysis patients.

**Methods:**

The randomized crossover trial in 48 patients compared MCO-Ci dialysis to High-flux dialysis of 4 weeks duration each plus 8 weeks extension phase. Primary endpoint was the gene expression of TNF-α and IL-6 in peripheral blood mononuclear cells (PBMCs), secondary endpoints were plasma levels of specified inflammatory mediators and cytokines.

**Results:**

After four weeks of MCO-Ci the expression of TNF-α mRNA (Relative quantification (RQ) from 0.92 ± 0.34 to 0.75 ± 0.31, -18.5%, p<0.001)-α and IL-6 mRNA (RQ from 0.78 ± 0.80 to 0.60 ± 0.43, -23.1%, p<0.01) was reduced to a significantly greater extent than with High-flux dialyzers (TNF mRNA-RQ: -14.3%; IL-6 mRNA-RQ: -3.5%). After retransformation of logarithmically transformed data, measurements after MCO were reduced to 82% of those after HF (95% CI 74%–91%). 4 weeks use of MCO-Ci resulted in long-lasting change in plasma levels of several cytokines and other substances with a significant decrease for sTNFR1, kappa and lambda free light chains, urea and an increase for Lp-PLA2 (PLA2G7) compared to High-flux. Albumin levels dropped significantly after 4 weeks of MCO dialysis but increased after additional 8 weeks of MCO dialysis. Twelve weeks treatment with MCO-Ci was well tolerated regarding the number of (S)AEs. In the extension period levels of CRP, TNF-α-mRNA and IL-6 mRNA remained stable in High-flux as well as in MCO-Ci.

**Conclusions:**

MCO-Ci dialyzers modulate inflammation in chronic HD patients to a greater extent compared to High-flux dialyzers. Transcription of pro-inflammatory cytokines in peripheral leukocytes is markedly reduced and removal of soluble mediators is enhanced with MCO dialysis. Serum albumin concentrations stabilize after an initial drop. These results encourage further trials with longer treatment periods and clinical endpoints.

## Introduction

The uremic syndrome is characterized by retention of a large number of solutes that are normally eliminated by the kidney [1, 2]. Many of these uremic toxins are not cleared by standard extracorporeal treatments, either because they are protein-bound or because their molecular weight exceeds the pore size of the membrane used. The latter group is often called middle molecules, consisting of compounds between 15 and 45 kDa such as β-2-microglobulin, tumor-necrosis factor alpha (TNF-α), interleukins, complement factor D and others, many of which are pro-inflammatory [1]. Although adverse effects or even toxicity has not been proven for all of these substances, many of them are factors or at least indicators of unfavorable outcome. For instance, an independent association between β-2-microglobulin levels and cardiovascular events was confirmed for different stages of chronic kidney disease (CKD) [3].

The incidence of cardiovascular disease in patients with CKD is very high and associated with the retention of uremic solutes [1]. Efficient renal replacement therapies that remove uremic toxins might reduce the high morbidity and mortality in end-stage renal disease (ESRD) patients. Proving a clinical advantage of more efficient dialysis techniques has been difficult. A benefit of High-flux dialysis compared to Low-flux dialysis with regards to all-cause mortality could not be clearly shown in the HEMO trial [4], and the MPO study demonstrated a survival advantage of High-flux only in subgroups (patients with low albumin and in diabetics) [5]. Hemodiafiltration (HDF) in post-dilution mode provides even more efficient removal of uremic retention solutes compared to High-flux [6]; however, the debate on improvements of clinical endpoints by HDF is still ongoing: three recent prospective randomized studies gave conflicting results regarding the benefit of HDF on mortality [7]^,^[8, 9]. All three studies reported nevertheless a dose-response relationship with the largest benefit in the subgroups with the highest substitution rates indicating that more efficient removal of uremic toxins might be beneficial.

To increase clearance of middle-sized molecules, highly permeable high cut-off dialyzers have been developed [10]. In a previous clinical trial we showed that treatment reduced multiple immune mediators in plasma (e.g. sTNFR1 by the use of these membranes [11]) and influences in vitro vascular calcification.[12] However, the routine treatment of chronic dialysis patients with HCO membranes is limited by significant losses of albumin [13]. Recently, a new membrane type with a steeper and lower cut-off, called medium cut-off membrane (MCO-Ci) has been developed [14]. The tailored pore sizes of these MCO-Ci membranes promote removal of large toxins while ensuring the retention of albumin. In a preceding clinical trial, the characteristics of MCO membranes were examined in single-session and it has been shown that these dialyzers provide improved clearance for middle molecules compared to High-Flux dialysis and High-Flux-Hemodiafiltration. [15]

Here, we report the results of a clinical trial using the MCO-Ci dialyzer for up to 12 weeks in a randomized, prospective, cross-over design in 50 chronic dialysis patients. This trial was conducted with the aim to examine the effects of MCO dialysis on markers of inflammation and uremic toxins.

## Materials and Methods

### Patients

**Fig 1:**
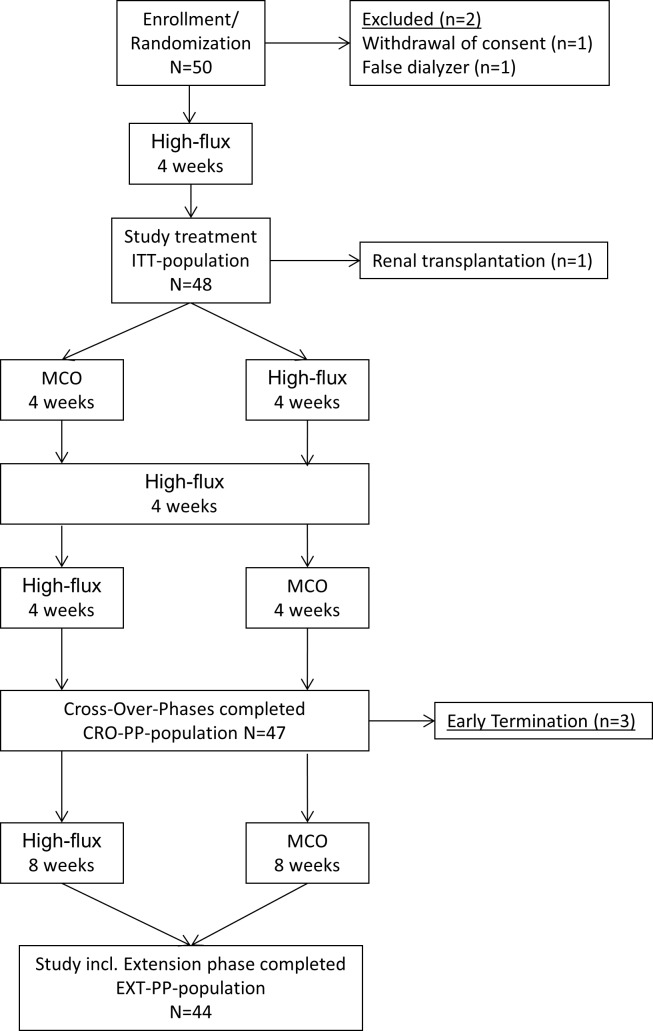
Patient Flow Chart.

This trial was a randomized, open-label, cross-over study. 50 chronic hemodialysis patients were randomized at two outpatient dialysis centers. (KfH dialysis centers at University of Halle/Saale and Charité Campus Virchow University Hospital Berlin, Germany) The first patient was included February 10th 2014, the study was finished July 29th 2014. The intention to treat population consisted of 48 patients while 47 completed the cross-over phase and 44 patients the extension phase per protocol (Fig 1).

Inclusion criteria included age between 18 and < 99 years and CKD G5 (GFR <15ml/min/1.73m^2^) requiring hemodialysis treatment thrice weekly since at least three months. Patients had to have a permanent vascular access (fistula, graft or a tunneled central venous catheter). Participation in the trial was impossible, if patients were unable or unwilling to give informed written consent, or were pregnant or breast feeding, had a clinically manifest infection or a CRP value of over 50 mg/l in the last two weeks. Moreover, therapy with immunosuppressive drugs and concurrent participation in another interventional study were exclusion criteria.

### Study treatment

The study was registered at ClinicalTrials.gov (NCT02084381). The study was conducted in accordance with the ethical principles of the Declaration of Helsinki and approved by the Ethics Committees of the Martin-Luther-University Halle-Wittenberg and the Charité, Berlin.

After informed written consent, the patients were randomized to one of two treatment regimens at a 1:1 ratio (block randomization with site as stratification factor). The random allocation was generated from a table of random numbers kept at the ‘The Coordination Center for Clinical Studies (KKS), Halle. The allocation code was transmitted to study physicians by fax. The trial was not blinded neither to patients nor to physicians, while laboratory staff remained blinded until data was cleared and the database closed.[11]

Throughout the whole trial all patients were treated with hemodialysis, hemodiafiltration was not conducted.

During the run-in phase of 12 dialysis sessions all patients were treated with High-flux dialyzers (Revaclear 400, Gambro Dialysatoren GmbH, Hechingen, Germany). Revaclear 400 dialyzers were used when High-flux is declared. Group A continued on this membrane while group B was switched to a MCO dialyzer prototype (MCO-Ci 400®, Gambro Dialysatoren GmbH, Table 1).[14]

**Table 1:** Study dialyzers.

Dialyzer	Mem-brane polymer	Membrane type	Fiber length(mm)	Fiber inner diameter (μm)	Membrane area (m^2^)	Membranewall thickness (μm)	UF coefficient (ml/h/ mm_Hg_)	Sterilization
MCO-Ci 400	PAES/PVP	medium cut-off (MCO 4)	236	180	1.8	35	50	Steam
Revaclear 400	PAES/PVP	high-flux (Poracton^TM^)	236	190	1.8	35	54	Steam

PAES, polyarylethersulfone; PVP, polyvinylpyrrolidone; UF, ultrafiltration.

The dialysis regimen of each patient including blood flow, dialysate flow and treatment time duration per session was not altered. After 12 treatments (4 weeks) a 4-week wash-out phase using High-flux dialyzers was performed to minimize carry-over-effects. Afterwards, patients crossed over to the other dialyzer for 4 weeks. Subsequently, to examine long-term effects, patients were treated for another 8 weeks with the dialyzer they were assigned to in the previous phase (Fig 1). The total duration of the study including the initial run-in phase was therefore 24 weeks. All patients were treated with bicarbonate dialysate of ultrapure quality; dialyzers were used only once. Blood samples were drawn prior to the dialysis sessions at the beginning and at the end of each period. At the beginning of each cross-over period, blood samples were also drawn before the first dialysis session. There were no changes to the protocol during the conduct of the study.

### Primary and secondary endpoints

Based on results from earlier clinical trials conducted with HCO dialyzers [11] we chose to examine the pre-dialysis TNF-α and IL-6 mRNA expression level in peripheral blood mononuclear cells (PBMC) before and after each 4-week cross-over period as the primary endpoint.

Secondary endpoints were plasma concentrations of multiple pro- and anti-inflammatory mediators.

### Laboratory analysis

#### Gene transcription analysis of TNF-α and IL-6

Gene transcription analysis of TNF-α and IL-6 were conducted as described earlier. [11] Briefly, the blood samples were collected into Tempus™ Blood RNA Tubes containing RNAse inhibitor (ThermoFisher Scientific) and immediately frozen at -20°C. Total RNA was extracted with Tempus™ Spin RNA Isolation Kit (ThermoFisher Scientific), followed by reverse transcription (500 ng of total RNA) with High Capacity cDNA Reverse Transcription Kit (Life Technologies). Amplification of all qPCR products was conducted in MicroAmp® 96-well plates (Life Technologies) by employment of StepOnePlus™® Real-Time PCR System (Life technologies) and following TaqMan Assays: TNF-α (Hs01113624_g1), IL-6 (Hs00985639_m1), ACTB (Beta actin, Hs99999903_m1) and RPL37A (Ribosomal Protein L37A, Hs99999903_m1, all from Life technologies). Thermal cycling conditions were performed according to manufacturer’s instructions.

For evaluation purposes, TNF-α and IL-6 expression was normalized with two housekeeping genes (ACTB and RPL37A) and divided by internal calibrator to obtain the fold of difference value (arbitrary unit for mRNA level) in comparison to the reference (positive control). For data evaluation, DataAssist Software (Life Technologies) was employed.

#### Soluble markers

Immune mediators were analyzed from citrate plasma using the commercially available Milliplex human Cytokine assay system (Millipore) to detect 19 proteins (GM-CSF, GRO, INF-γ, IL-10, IL-12(p40), IL-12(p70), IL-17A, IL-1β, IL-1ra, IL-2, IL-4, IL-6, IL-8, MCP-1, MIP-1α, MIP-1β, sCD40L, sIL2-Ra, TNFα); FGF23, Insulin and Leptin were measured using the Milliplex human bone panel. A multiplexed Luminex-based immunoassay for soluble receptor proteins (ICAM, RAGE, VCAM, MIF, gp130, sTNF-R1, sTNF-R2, Fas, E-Selectin, sIL6R), Rantes, MIF, and Lipoprotein-associated phospholipase A2 (PLA2) was developed and validated in-house as described before by Hsu et al.[16]

For quantification of interleukin-6 (IL-6) the Quantikine HS human IL-6 ELISA kit, for complement factor D (CFD) the human CFD ELISA kit, for uPAR the human uPAR ELISA kit (R&D Systems, Minneapolis, USA); for α1-microglobulin (α1M) the 1M ELISA kit (Abcam, Cambridge, UK); for YKL-40 the MicroVue YKL-40 ELISA kit (Quidel, San Diego, USA) were used according to manufacturers’ instructions.

Kappa and lambda free light chains (FLC), β-2-microglobulin and myoglobin were quantified by nephelometry on a Siemens BN Prospec Analyzer (Siemens, Marburg, Germany) with Freelite FLC detection kits (The Binding Site, Birmingham, UK) or BN Prospec System reagent kits for β-2-microglobulin or myoglobin (Siemens, Marburg, Germany) as previously described [17–19].

Serum albumin, CRP, urea, creatinine, electrolytes, hemoglobin and platelets were measured at the central laboratories of the local university clinics.

### Statistics

The design of the study, crossover with extension of second period, required modified definitions of study populations. The two populations used in our analysis were

(Modified) ITT-CRO Population (Intent To Treat crossover phase): Includes all patients with measurements from at least one period of the crossover part and without clinical events which make measurement of primary endpoint impossible.

(Modified) ITT-EXT Population (Intent To Treat crossover phase plus extension phase): Includes all patients which are ITT-CRO and which contribute measurements from at least one visit of the extension part and which had no clinical events which make measurement of the primary endpoint impossible during the extension part.

The complete definitions of populations are given in the supplement.

The primary analysis population is the modified ITT-CRO population. Patients with clinical events making the analysis of the primary endpoint impossible were excluded after a blind data review. The remaining drop outs were included in the analysis if results were available for at least one treatment period.

Assessment of Populations: Populations were assessed in a blind data review. In this review, data were blinded for treatment (experimental, control). Blinding for periods (time sequence) was not possible, as study deviations only appeared in crossover period 2 or during the extension part of the study. The criteria for assessing a specific subject in an analysis population were predefined with the option of modification during the casuistic assessment process.

Categorical variables are presented as percentage of patients. Continuous data are expressed as means and standard deviation or median, interquartile range where appropriate. Quantitative variables were assessed for normal distribution. If necessary, log transformations were applied. In general, the log transformation provided acceptable fit to normal distribution for the lab values and vital signs under investigation. Linear models with generalized estimating equations (GEE) were used for baseline adjusted intra-individual comparisons. The model contained baseline as continuous covariate for each crossover phase, treatment and time as binary factors. The exchangeable correlation structure was chosen. The primary analysis referred to the null hypothesis “treatment parameter = 0”. The sample size estimation was done for a simple t-test, based on an effect size of 0.5 (as seen in a preceding study[11]) and a drop out rate of 30%.

Imputation of missing values was not performed.

The level of significance was 0.05 (two-sided) but only the primary endpoint was tested confirmatory. P-values in secondary analyses serve descriptive purposes. All analyses were done using SPSS for Windows (release 21) at the Institute for Clinical Epidemiology and Applied Biometry, University of Tübingen.

## Results

Table 2 gives the demographic data of the ITT-CRO population which included 48 patients. There were no significant differences between the patients randomized to MCO first or to High-flux first. Fig 1 denotes the patients’ flow chart during the trial periods.

**Table 2:** Demography and Clinical Patient Data ITT Population.

	MCO first (n = 23)	HF first (n = 25)	p-value
**Demography**			
Patients Age (years)	58.1±16.6	59.8±16.5	0.72
Male Patients	n = 19 (83%)	n = 16 (64%)	0.2
Female Patients	n = 4 (17%)	n = 9 (36%)	
Patients at Centre Berlin	n = 11 (48%)	n = 13 (52%)	0.77
Patients at Centre Halle	n = 12 (52%)	n = 12 (48%)	
**Anthropometry**			
Body Height (cm)	173.6 (±8.6)	171.5 (±7.4)	0.38
Body Weight (kg)	83.3 (±13.7)	79.2 (±15.0)	0.58
BMI (kg/m^2^)	27.6 (±4.1)	27.1 (±5.4)	0.66
**Medical History**			
Dialysis vintage (months)	77.3 (±78)	54.0 (±50)	0.35 (Mann Whitney)
Venous access*			
AV Fistula	21	24	0.42 (Monte Carlo)
Graft	2	1	
Tunneled catheter	2	0	
**Underlying Renal Disease**			
Diabetic nephropathy	3 (13)	5 (20)	0.093 (Monte Carlo simulation used because of small cell frequencies)
Glomerulonephritis	4 (17)	5 (20)	
Interstitial, Analgetics, Reflux	3 (13)	2 (8)	
Cysts	1 (4)	5 (20)	
Hypertension, Glomerulosclerosis	6 (26)	0 (0)	
Others	6 (26)	7 (28)	
Unknown	0 (0) 1 (4)		
**Screening/Baseline**			
Diastolic blood pressure (mmHg)	78.2 (±12.7)	75.7 (±13.0)	0.51
Systolic blood pressure (mmHg)	140.0 (±22.0)	141.2 (±23.4)	0.84
Blood flow (ml/min)	299 (±38,4)	299 (±30,8)	0,55
Dialysis time/session (min)	267 (±21,2)	263 (±18,992)	0,76
Total UF (ml)	2304 (±932)	1926 (±942)	0.17
**Residual urine output**			
0–499 ml/d	16 (70)	14 (56)	0.38
>500ml/d	7 (30)	11 (44)	
**Anticoagulation**			
Heparin-fractionated	5 (22)	3 (12)	0.45
Heparin non-fractionated	18 (78)	22 (88)	

Abbreviation: MCO = medium cut-off; HF = high flux, Entries are absolute and percentage frequencies and means ± Standard Deviations* P-values refer to two-sided t-tests and chi-square tests if not stated explicitly.

*Discrepancy caused by change of access during trial.

### Primary endpoint

**Fig 2:**
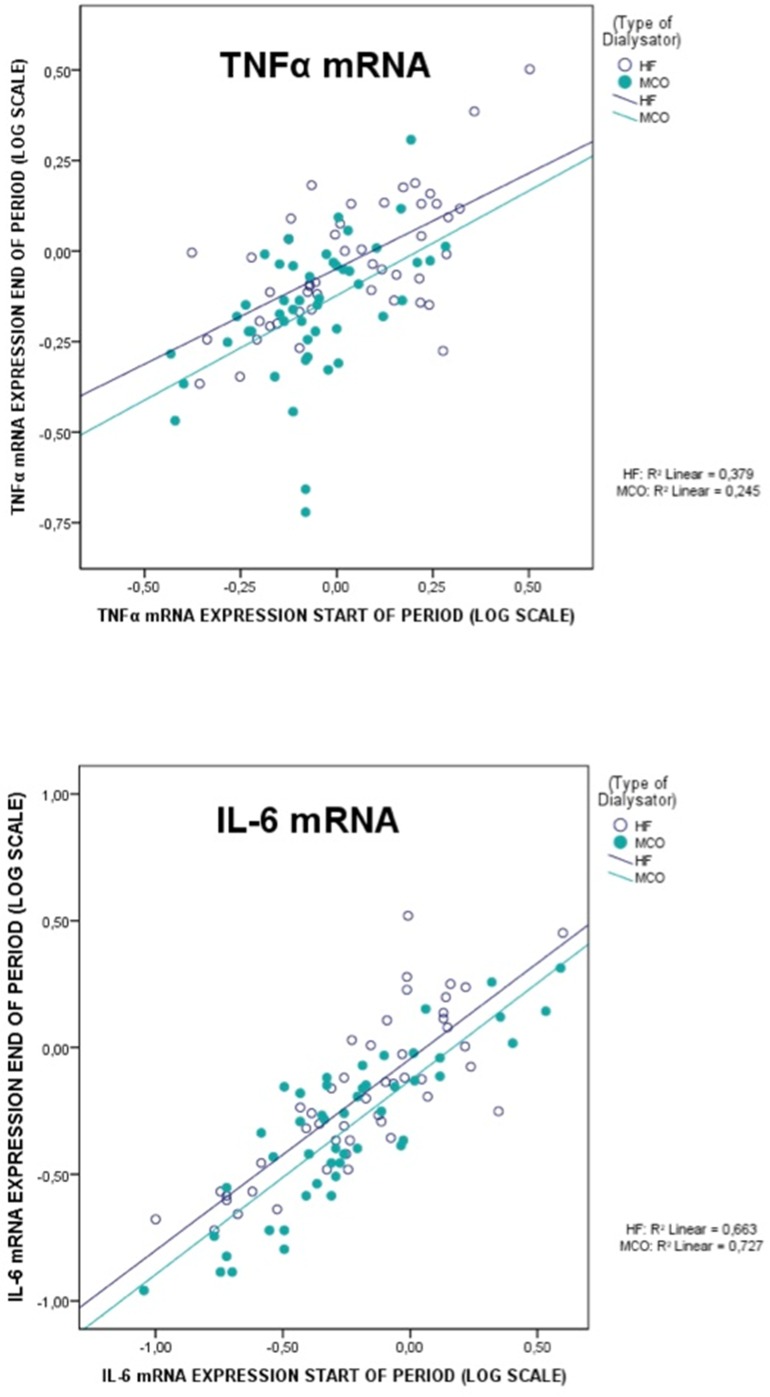
Expression of TNF-mRNA and IL-6-mRNA (log scale of arbitrary units) before and after the cross-over periods using High-flux or MCO dialyzers. Each dot (MCO: solid; Highflux: open) represents a single patient. Note that the regression line of the MCO phase is lower and does not cross the line of the High-flux period; the difference between High-flux and MCO is significant, p<0.01.

The primary endpoint, TNF-α mRNA and IL-6 mRNA transcripts, was significantly different between MCO-Ci and High-flux (Table 3). Both transcripts were downregulated at the end of the crossover treatment period with High-flux as well as with MCO-Ci. However, MCO-Ci reduced the expression of TNF-α mRNA as well as IL-6 mRNA to a larger extent as compared to HF. After retransformation of logarithmically transformed data, measurements after MCO were reduced to 82% of those after HF (95% CI 74%–91%). Fig 2 illustrates the dialyzer effect on gene transcripts for each individual patient.

**Table 3:** Primary endpoints (TNF- α mRNA / IL-6 mRNA) and long-term plasma levels before and after 4 weeks treatment with High-flux / MCO. Shown are only parameters with a significant change after the MCO-period.

	High-flux		MCO		p MCO vs HF
	T = 0	T = 4 weeks	T = 0	T = 4 weeks	
Primary endpoint					
TNF-α mRNA	1.19 ± 0.57	1.02 ± 0.49*	0.92 ± 0.34	0.75 ± 0.31**	< 0.001
IL-6 mRNA	0.86 ± 0.68	0.83 ± 0.67	0.78 ± 0.80	0.60 ± 0.43**	0.001
Clinical chemistry					
Albumin g/l	36.6 ± 3.2	37.5 ± 2.7	37.0 ± 3.6	35.3 ± 3.7**	< 0.001
CRP mg/l	13.4 ± 25.5	9.6 ± 15.7	15.3 ± 30.0	9.3 ± 14.5	n.s.
Urea mg/dl	131± 38	129 ± 35	128 ± 34	115 ± 29**	0.012
Beta2M mg/l	27.0 ± 9.1	26.1 ± 8.6	26.9 ± 8.4	25.7 ± 8.1**	n.s.
TNF-/TNFR-Family					
sTNF-R1 ng/ml	13.3 ± 4.7	12.9 ± 4.7	13.0 ± 4.4	11.0 ± 3.7**	0.01
sTNF-R2 ng/ml					
TNF-α pg/ml	23.4 ± 7.3	22.2 ± 6.0	24.1 ± 8.1	20.6 ± 5.8**	n.s.
sCD40 pg/ml	2238 ± 1705	2044 ± 1382	2403 ± 1980	1867 ± 1297*	n.s.
Main cytokines					
IFN-γ pg/ml	16.0 ± 19.7	14.4 ± 17.8	17.5 ± 19.2	11.8 ± 12.7**	n.s.
IL-17 pg/ml	7.2 ± 7.9	6.5 ± 6.9	8.9 ± 9.5	5.4 ± 5.6**	n.s.
IL-10 pg/ml	59 ± 355	47 ± 280	51 ± 287	65 ± 402**	n.s.
IL-12p40 pg/ml	22.8 ± 16.4	25.4 ± 24.0	26.9 ± 23.1	21.3 ± 17.3*	n.s.
IL-6 pg/ml	9.8 ± 20.5	5.5 ± 4.5*	9.0 ± 13.2	6.0 ± 5.9**	n.s.
Chemotaxis/Adherence					
IL-8 pg/ml	11.7 ± 8.5	10.9 ± 7.9	13.0 ± 9.5	10.8 ± 7.8*	n.s.
MCP-1 pg/ml	480 ± 219	466 ± 152	492 ± 166	444 ± 151*	n.s.
MIP-1b pg/ml	24.8 ± 15.3	22.8 ± 13.6	26.9 ± 15.5	22.1 ± 11.8**	n.s.
sVCAM ng/ml	166 ± 31	150 ± 43**	163 ± 41	149 ± 32*	n.s.
Other					
FLC kappa mg/l	134 ± 65	140 ± 77	137 ± 65	120 ± 54**	0.003
FLC lambda mg/l	91 ± 42	91 ± 44	95 ± 46	79 ± 36**	< 0.001
Fetuin A μg/ml	569 ± 124	543 ± 122	560 ± 131	519 ± 112*	n.s.
Lp-PLA2 ng/ml	180 ± 90	185 ± 108	156 ± 76	189 ± 101**	0.026

* = p<0.05 vs. T = 0

** = p<0.01 vs. T = 0.

No interaction terms with treatment were significant. Thus, analyses stratified for baseline or treatment period were not necessary. However, for the primary endpoint, the different study centers were analyzed separately. Results were comparable to the overall analysis. For both Halle (p < 0.001) and Berlin (p = 0.010) MCO-Ci revealed significantly lower expression of TNF-α mRNA as compared to HF. A time effect (crossover period 2 > crossover period 1) was significant only in Berlin (p < 0.001), but not in Halle (p = 0.168). Baseline was significantly correlated with outcome in both study centers. In summary, the results in the study centers were coherent and support the primary analysis indicating superiority of MCO-Ci compared to High-flux. Further sensitivity and subgroup analyses showed that this difference is primarily borne by crossover period 1 (even so time treatment interaction was not significant) and that there seem to be differences between study centers Halle and Berlin.

Additionally, an analysis combining the second crossover period 2 with the extension period (12 weeks in parallel group design ignoring crossover period 1) was done. However, here no significant difference was seen between the two membranes for TNF-α mRNA nor IL-6 mRNA.

### Secondary endpoints

During the trial a broad panel of immune-modulatory plasma proteins were measured. The plasma levels before and after the 4 weeks cross-over phases are listed in Table 3. Listed are only those parameters that changed significantly during the MCO-Ci phase. All other parameters did not change significantly. Many of the reported parameters also changed during the High-flux period; a significant difference between MCO-Ci and High-flux dialyzers was observed only for sTNF-R1, kappa and lambda free light chains, albumin and urea (decrease during MCO-Ci) and Lp-PLA2 (increase during MCO-Ci).

Serum albumin was influenced by the MCO-Ci membrane; however, no albumin substitution was necessary in any of the participating patients. There was a significant decrease in serum albumin in the MCO-Ci phase (from 37.0±3.6 to 35.3±3.7 g/L, p<0.01) while albumin remained stable in the High-flux phase (baseline: 36.6±3.2, 4 weeks: 37.5±2.7 g/L).

### Extension phase

Primary endpoints and safety-related endpoints were obtained before and after the extension phase in which patients were dialyzed for additional 8 weeks with the assigned dialyzer. As shown in Table 4, serum albumin was stable after 8 weeks with High-flux (37.6 to 37.9 g/l), and increased non-significantly after 8 weeks with MCO-Ci (35.7 to 36.4 g/l). TNF-α mRNA and IL-6-mRNA remained stable over the course of this 12-week phase Table 4).

**Table 4:** Primary endpoints and plasma levels of Albumin, Free light chains and soluble TNF receptor 1 during the second crossover phase (4 weeks) and the extension phase (8 weeks).

	High-Flux			MCO			P (MCO vs Highflux, Start phase 2—End phase 3)
	Start Phase 2	End Phase 2	End Phase 3	Start Phase2	End Phase 2	End Phase 3	
TNF-alpha mRNA RQ	1.06 (±0.62)	1.06 (±0.67)	1.03 (±0.43)	0.85 (±0.33)	0.80 (±0.25)	0.87 (±0.31)	0,702
IL-6 mRNA RQ	0.91 (±0.82)	1.00 (±0.85)	0.89 (±0.73)	0.52 (±0.26)	0.53 (±0.29)	0.62 (±0.42)	0,414
Kappa FLC mg/L	147.83 (±64.35)	150.39 (±76.33)	144.51 (±71.07)	140.76 (±68.72)	117.03 (±55.53)	113.96 (±54.95)	0,001
Lambda FLC mg/L	90.97 (±33.02)	93.92 (±38.92)	89.61 (±36.39)	96.28 (±56.14)	80.50 (±43.97)	75.98 (±37.89)	<0,0001
sTNFr-1 ng/mL	13.09 (±3.7)	12.605 (±4.6)	12.99 (±4.9)	12.76 (±4.68)	10.81 (±4.39)	10.44 (±3.65)	0,006
Albumin g/L	35.98 (±2.99)	37.6 (± 2.3)	37.86 (±3.47)	37.16 (±4.08)	35.7 (±4.5)	36.41 (±3.89)	<0,0001

Treatment with the MCO-Ci dialyzer was generally well tolerated. 16 serious adverse events (SAE) occurred throughout the trial (Table 5). 4 SAE arose while the patients were treated with High-flux dialyzers and 6 while treated with the MCO-Ci dialyzer. 4 SAE were classified as severe, of these 3 during High-flux period and 1 during the washout period after cross-over periods. No SAE was classified as related to the study product. In 11 of the 16 SAEs, patients fully recovered, one death occurred during the High-flux period (Table 5).

**Table 5:** Number of patients with serious adverse events while being treated in the study (+ 15 days after end of study). (ITT population, n = 48).

Serious adverse events	Before first dialysis	During High-flux Treatment	During MCO Treatment	Washout Phase after Treatment	Total
SAE	2	4	6	3	15
Severe SAE	0	3	0	1	4
SAE Related to Study Product	0	0	0	0	0
Study Product permanently stopped	0	1	0	0	1
Fully Recovered	1	3	5	2	11
Death	0	1	0	0	1

## Discussion

The present clinical trial examined the middle-term effects of the newly developed MCO-Ci membranes. Our results show that both TNF-α and IL-6 mRNA are lowered to a greater extent with MCO-Ci compared to High-flux. This underlines the capacity to reduce the inflammatory level in chronic dialysis patients with MCO-Ci dialysis.

We chose TNF-α and IL-6 mRNA in blood leukocytes as the primary endpoint for a variety of reasons. First, in a previous trial using HCO dialyzers we found that gene expression in peripheral blood is a reliable marker to study inflammation in hemodialysis patients [11]. Second, interpretation of the measured concentrations of serum parameters depends on several factors. Binding to other proteins such as soluble receptors, formation of multimers, distribution and re-shift from tissue and other factors influence serum concentrations of interleukins and makes their measurement and interpretation difficult. MRNAs of inflammatory parameters such as IL-6 and TNF-α on the other hand are intracellular markers and are thus not eliminated during dialysis.

Intracellular inflammatory parameters are hence better suited than plasma levels as markers of microinflammation.

Besides the significant reduction of the mRNAs the serum concentrations of TNF-α and IL-6 were also reduced significantly with MCO but the reduction was not significant when compared to High-Flux. IL-6 and TNF-α are important inflammatory proteins in the process of inflammation and correlate with cardiovascular events in the dialysis population. [20–22]

Other interleukins that were significantly reduced with MCO-Ci included sTNFR1. In accordance with the results of the PERCI-I-trial, levels of sTNFR1 were lowered significantly better with MCO-Ci dialysis compared to High-Flux. sTNFR1 is known as a predictor of cardiovascular mortality in chronic dialysis patients [23]. It seems possible, that the drop of this 18 kD -molecule was partially caused by improved elimination. However, since we did not measure sTNFR1 concentrations in dialysate samples other factors such as reduced sTNFR1 production as a result of generally lowered microinflammation may also have played a role.

On a regulatory point of view the TNF-/TNFR superfamily seems to play an important role in this setting. It fits in the line that sTNFR1 which is believed to have a dual role–(firstly sTNFR acts as a neutralizing and buffering component for circulating TNF-α secondly the soluble receptor may induce apoptosis via reverse TNF-α-signaling)—is significantly decreased by MCO-Ci-treatment.

After four weeks of MCO-Ci dialysis Interleukin-10 and Lp-PLA2 increased significantly. While IL-10 is known as antiinflammatory, the role of Lp-PLA2 in the process of atherosclerosis is poorly understood and controversely discussed in the literature [24–26].

Several other molecules were significantly changed after MCO-Ci dialysis, but lacked significance when compared to high-flux dialysis (see Table 3). Taken together, the MCO-Ci dialyzer appears to influence the inflammatory signature of immune regulatory lymphocytes and monocytes, as most of the main inflammatory cytokines of both leukocyte subsets are downregulated (Table 3).

Besides modulation of inflammation we examined the course of albumin concentrations with MCO-Ci dialysis. Our results demonstrate that in spite of the steeper cut-off compared to High Cut-off membranes loss of albumin remains an important issue in patients treated with MCO dialysis. In accordance with the first clinical trial in which MCO dialyzers came to use we noted that with the use of MCO-Ci filters the drop in in mean albumin concentration was less than 2 g/L and thus albumin loss seems to be limited compared to HCO.[11] We did not conduct systematic measurements of albumin concentrations in the dialysate. But considering the higher cut-off of the membrane it seems highly likely that lower albumin concentrations after MCO dialysis is brought about by increased albumin filtration and/or membrane adherence.

Extending the use of MCO for another 8 weeks did not result in further reductions in serum albumin but even increased again.

Moreover, the number of clinically overt side effects was similar in both treatment arms and no AE related to hypalbuminemia was reported. During both the two 4-weeks period and the 8-weeks extension period SAEs were equally distributed in both treatment arms without statistical significant differences.

Still, the extent of albumin loss has to be considered as low albumin levels are associated with negative long-term adverse outcomes due to malnutrition. This is an important aspect that has to be evaluated in future trials.

Thus, trials with longer lasting treatment periods and clinical endpoints are desirable to analyze this matter further.

The present study has a number of advantages over previous trials on this matter. It investigated the middle-term effects of dialyzers on cytokine levels and inflammatory parameters. Most previous studies on HCO and MCO only examined short term effects on plasma levels or dialyzer clearances.[27]

### Limitations

By design, our trial focuses on surrogate parameters. Therefore, no statements can be made regarding clinical endpoints. Furthermore, we only used both dialyzers in a diffusive mode and cannot state whether MCO-Ci dialysis is superior to HDF regarding the elimination of middle-sized molecules.

Also, the study was an open-label study only blinded to staff performing the lab analysis and the data review, but not to patients and dialysis nurses and doctors. Even though this trial was monitored carefully, this may possibly have interfered with the interpretation of filter tolerance and (S)AEs.

Moreover, we saw a difference regarding the results during both treatment periods and in the extension period. While there was a great difference in the primary endpoint with respect to the dialyzer in the first phase, no significant difference was noted in the second phase and the extension period. Even though no significant carryover effect was noted in the statistical analysis, it can be suspected that the MCO treatment caused longer lasting effects than expected. In the group that started with MCO, possibly the preceding treatment with MCO may have interfered with the results from the second phase using HF. In future trials a mere parallel group design thus appears preferable.

Finally, cytokine or albumin removal was not analyzed by collecting complete spent dialysate. We decided against this option because most cytokines in blood are at a very low concentration range of pg/mL that will even be lower in diluted dialysate. Cytokine concentrations in dialysate samples would therefore be below the detection limit of most assays. Furthermore, possibly a significant portion may be lost via membrane adherence and thus would also be missed with analysis of the dialysate.

It therefore, remains somewhat unclear whether the effects on serum concentrations of the investigated parameters are due to reduced production or enhanced elimination.

In summary, we demonstrated a trial with MCO-Ci membranes with treatment periods of up to twelve weeks. The use of MCO-Ci reduced the primary endpoint TNF-α and IL-6 mRNA expression in peripheral blood to a significantly greater extent compared to High-flux dialyzers. 4 weeks use of MCO-Ci resulted in a persistent change in plasma levels of several cytokines and other substances with a significant difference to High-flux for sTNFR1, kappa and lambda free light chains, albumin, urea and Lp-PLA2. While the present trial focuses on dampening chronic inflammation in dialysis patients, the results also suggest MCO-Ci dialysis as a treatment option for other disease entities demanding removal of solutes in this molecular size range such as free light chains, myoglobin, and cytokines in sepsis. Thus, possible indications that have to be assessed in future trials may include sepsis, multiple myeloma and rhabdomyolysis.

## Supporting Information

S1 File Clinical Study Protocol. (PDF)

S2 File Consort checklist. (PDF)

S3 File Supplementary statistic data. (PDF)

## References

[1] Duranton F, Cohen G, De Smet R, Rodriguez M, Jankowski J, Vanholder R, et al Normal and pathologic concentrations of uremic toxins. J Am Soc Nephrol. 2012;23(7):1258–70. PubMed Central PMCID: PMC3380651. 10.1681/ASN.2011121175 22626821 PMC3380651

[2] Vanholder R, De Smet R, Glorieux G, Argiles A, Baurmeister U, Brunet P, et al Review on uremic toxins: classification, concentration, and interindividual variability. Kidney international. 2003;63(5):1934–43. 10.1046/j.1523-1755.2003.00924.x 12675874

[3] Liabeuf S, Lenglet A, Desjardins L, Neirynck N, Glorieux G, Lemke HD, et al Plasma beta-2 microglobulin is associated with cardiovascular disease in uremic patients. Kidney international. 2012;82(12):1297–303. 10.1038/ki.2012.301 22895515

[4] Eknoyan G, Beck GJ, Cheung AK, Daugirdas JT, Greene T, Kusek JW, et al Effect of dialysis dose and membrane flux in maintenance hemodialysis. The New England journal of medicine. 2002;347(25):2010–9. 10.1056/NEJMoa021583 12490682

[5] Locatelli F, Martin-Malo A, Hannedouche T, Loureiro A, Papadimitriou M, Wizemann V, et al Effect of membrane permeability on survival of hemodialysis patients. Journal of the American Society of Nephrology: JASN. 2009;20(3):645–54. PubMed Central PMCID: PMC2653681. 10.1681/ASN.2008060590 19092122 PMC2653681

[6] Meert N, Eloot S, Waterloos MA, Van Landschoot M, Dhondt A, Glorieux G, et al Effective removal of protein-bound uraemic solutes by different convective strategies: a prospective trial. Nephrology, dialysis, transplantation: official publication of the European Dialysis and Transplant Association—European Renal Association. 2009;24(2):562–70. 10.1093/ndt/gfn522 18809977

[7] Ok E, Asci G, Toz H, Ok ES, Kircelli F, Yilmaz M, et al Mortality and cardiovascular events in online haemodiafiltration (OL-HDF) compared with high-flux dialysis: results from the Turkish OL-HDF Study. Nephrol Dial Transplant. 2013;28(1):192–202. 10.1093/ndt/gfs407 23229932

[8] Maduell F, Moreso F, Pons M, Ramos R, Mora-Macia J, Carreras J, et al High-efficiency postdilution online hemodiafiltration reduces all-cause mortality in hemodialysis patients. Journal of the American Society of Nephrology: JASN. 2013;24(3):487–97. PubMed Central PMCID: PMC3582206. 10.1681/ASN.2012080875 23411788 PMC3582206

[9] Grooteman MP, van den Dorpel MA, Bots ML, Penne EL, van der Weerd NC, Mazairac AH, et al Effect of online hemodiafiltration on all-cause mortality and cardiovascular outcomes. Journal of the American Society of Nephrology: JASN. 2012;23(6):1087–96. PubMed Central PMCID: PMC3358764. 10.1681/ASN.2011121140 22539829 PMC3358764

[10] Boschetti-de-Fierro A, Voigt M, Storr M, Krause B. Extended characterization of a new class of membranes for blood purification: the high cut-off membranes. Int J Artif Organs. 2013;36(7):455–63. 10.5301/ijao.5000220 23661558

[11] Girndt M, Fiedler R, Martus P, Pawlak M, Storr M, Boehler T, et al High cut-off dialysis in chronic hemodialysis patients. Eur J Clin Invest. 2015. 10.1111/eci.12559 26519693

[12] Zickler D, Willy K, Girndt M, Fiedler R, Martus P, Storr M, et al High cut-off dialysis in chronic haemodialysis patients reduces serum procalcific activity. Nephrology, dialysis, transplantation: official publication of the European Dialysis and Transplant Association—European Renal Association. 2016. 10.1093/ndt/gfw293 27445317

[13] Kneis C, Beck W, Boenisch O, Klefisch F, Deppisch R, Zickler D, et al Elimination of middle-sized uremic solutes with high-flux and high-cut-off membranes: a randomized in vivo study. Blood purification. 2013;36(3–4):287–94. 10.1159/000356224 24496201

[14] Boschetti-de-Fierro A, Voigt M, Storr M, Krause B. MCO Membranes: Enhanced Selectivity in High-Flux Class. Scientific reports. 2015;5:18448 PubMed Central PMCID: PMC4680880. 10.1038/srep18448 26669756 PMC4680880

[15] Kirsch AH, Lyko R, Nilsson LG, Beck W, Amdahl M, Lechner P, et al Performance of hemodialysis with novel medium cut-off dialyzers. Nephrology, dialysis, transplantation: official publication of the European Dialysis and Transplant Association—European Renal Association. 2016. 10.1093/ndt/gfw310 PMC5837492 27587605

[16] Hsu HY, Wittemann S, Schneider EM, Weiss M, Joos TO. Suspension microarrays for the identification of the response patterns in hyperinflammatory diseases. Medical engineering & physics. 2008;30(8):976–83. 18313970 10.1016/j.medengphy.2008.01.003

[17] Hutchison CA, Plant T, Drayson M, Cockwell P, Kountouri M, Basnayake K, et al Serum free light chain measurement aids the diagnosis of myeloma in patients with severe renal failure. BMC nephrology. 2008;9:11 PubMed Central PMCID: PMC2564915. 10.1186/1471-2369-9-11 18808676 PMC2564915

[18] Keir R, Evans ND, Hutchison CA, Vigano MR, Stella A, Fabbrini P, et al Kinetic modelling of haemodialysis removal of myoglobin in rhabdomyolysis patients. Computer methods and programs in biomedicine. 2014;114(3):e29–38. 10.1016/j.cmpb.2013.07.017 24008249

[19] Krieter DH, Devine E, Wanner C, Storr M, Krause B, Lemke HD. Clearance of drugs for multiple myeloma therapy during in vitro high-cutoff hemodialysis. Artificial organs. 2014;38(10):888–93. 10.1111/aor.12248 24392952

[20] Tintut Y, Patel J, Parhami F, Demer LL. Tumor necrosis factor-alpha promotes in vitro calcification of vascular cells via the cAMP pathway. Circulation. 2000;102(21):2636–42. 11085968 10.1161/01.cir.102.21.2636

[21] Al-Aly Z. Arterial calcification: a tumor necrosis factor-alpha mediated vascular Wnt-opathy. Translational research: the journal of laboratory and clinical medicine. 2008;151(5):233–9. 18433704 10.1016/j.trsl.2007.12.005

[22] Lu P, Liu J, Pang X. Pravastatin inhibits fibrinogen- and FDP-induced inflammatory response via reducing the production of IL-6, TNF-alpha and iNOS in vascular smooth muscle cells. Mol Med Rep. 2015;12(4):6145–51. 10.3892/mmr.2015.4149 26238934

[23] Neirynck N, Glorieux G, Schepers E, Verbeke F, Vanholder R. Soluble tumor necrosis factor receptor 1 and 2 predict outcomes in advanced chronic kidney disease: a prospective cohort study. PloS one. 2015;10(3):e0122073 PubMed Central PMCID: PMC4379033. 10.1371/journal.pone.0122073 25823004 PMC4379033

[24] Talmud PJ, Holmes MV. Deciphering the Causal Role of sPLA2s and Lp-PLA2 in Coronary Heart Disease. Arterioscler Thromb Vasc Biol. 2015;35(11):2281–9. 10.1161/ATVBAHA.115.305234 26338298

[25] Karabina S, Ninio E. Plasma PAFAH/PLA2G7 Genetic Variability, Cardiovascular Disease, and Clinical Trials. Enzymes. 2015;38:145–55. 10.1016/bs.enz.2015.09.002 26612651

[26] Katan M, Moon YP, Paik MC, Wolfert RL, Sacco RL, Elkind MS. Lipoprotein-associated phospholipase A2 is associated with atherosclerotic stroke risk: the Northern Manhattan Study. PLoS One. 2014;9(1):e83393 PubMed Central PMCID: PMC3886969. 10.1371/journal.pone.0083393 24416164 PMC3886969

[27] Haase M, Bellomo R, Baldwin I, Haase-Fielitz A, Fealy N, Morgera S, et al Beta2-microglobulin removal and plasma albumin levels with high cut-off hemodialysis. Int J Artif Organs. 2007;30(5):385–92. 17551901 10.1177/039139880703000505

