# Supplementary material for: Medium Cut-Off (MCO) Membranes Reduce Inflammation in Chronic Dialysis Patients—A Randomized Controlled Clinical Trial
Source: PLoS One. 2017 Jan 13;12(1):e0169024. doi: 10.1371/journal.pone.0169024 (PMC5234772; doi:10.1371/journal.pone.0169024)
Supplement: S1 File — (PDF) [file pone.0169024.s001.pdf]

**Title:**	***P**ermeability **E**nhancement to **R**educe **C**hronic **I**nflammation _ Medium cut off (MCO)*

Compliance with GCP:	To be Reviewed by German Ethics Committee and by Competent Authority
Study type:	The study will be conducted according to Medical Device current regulation
Phase	Pilot Study
Investigational product:	MCO-Ci 400
Comparator product:	Revaclear 400
Number of treatments:	72/patient
Number of patients:	50 randomized patients (25 patients/ randomization group)
Target population:	Hemodialysis (HD)
Study duration:	24 weeks (per patient)
Recruitment period:	two weeks (Period of time needed to include (not to enroll) the number of patient planned by the protocol)
Patient study period:	First Patient In (FPI): 2014-02-01<br>Last Patient Out (LPO): 2014-07-31
Study Registration number:	EUDAMED

TABLE OF CONTENTS

LIST OF AMENDMENTS 6
LIST OF ABBREVIATIONS 8
STUDY SCHEDULE 12
1. INTRODUCTION 12
1.1. BACKGROUND AND RATIONALE 12
1.2. BENEFITS AND RISKS FOR THE STUDY POPULATION 14
1.3. JUSTIFICATION OF TREATMENT PERIODS AND EVALUATION SCHEDULE 14
2. STUDY OBJECTIVE 15
2.1. PRIMARY OBJECTIVE 15
2.2. SECONDARY OBJECTIVES 15
3. STUDY PARAMETERS AND ENDPOINTS 15
3.1. PRIMARY ENDPOINTS 15
3.2. SECONDARY ENDPOINTS 15
4. STUDY DESIGN 16
4.1. STUDY DURATION 16
4.2. STUDY PERIODS 17
4.3. STUDY SUSPENSION AND DISCONTINUATION RULES 17
4.3.1. Procedure for resuming the clinical investigation after temporary suspension 17
4.4. PRODUCT AND MATERIAL 17
4.4.1. General description of Investigational Product 18
4.4.1.1. Manufacturing process 18
4.4.1.2. Investigational Product labeling 19
4.4.2. General description of Comparator Product 19
4.4.2.1. Manufacturing process 19
4.4.2.2. Comparator Product labeling 19
Each investigational device will bear the following information: 19
4.4.3. Other material 19
4.5. PRODUCT ACCOUNTABILITY 20
5. SELECTION AND WITHDRAWALS OF PATIENTS 20
5.1. STUDY-SPECIFIC DEFINITIONS 20
5.2. STUDY POPULATION 20
5.2.1. Vulnerable population 20
5.3. INCLUSION CRITERIA 20
5.4. - EXCLUSION CRITERIA 21
5.5. RECRUITMENT AND SAMPLE SIZE 21
5.6 PATIENT WITHDRAWAL AND REPLACEMENT 21
6. PATIENT TREATMENT 22
6.1. RECOMMENDATION FOR STUDY PRODUCTS IMPLEMENTATION 22
6.2. CONCOMITANT MEDICATION 22
6.2.1. Medication allowed 22
6.2.2. Medication not allowed 22
6.3. TREATMENT DETAILS 22
6.3.1. Dialysis treatment 22
6.3.2. Heparin strategy 22
6.4. PATIENT MEDICAL CARE AFTER CLINICAL STUDY CLOSURE 22
7. LABORATORY AND ASSESSMENT 22
7.1. STUDY VISITS 23
7.2. SAMPLING SCHEDULE 23
7.3. SAMPLING DETAILS 23

7.4. PARAMETERS AND LABORATORY ANALYZES ... 24
7.4.1. Local analyzes ... 24
7.4.2. Central analyzes ... 24
EVALUATED PARAMETERS AND METHOD ... 26

8. PATIENT PARAMETERS TO BE RECORDED ON CRF ... 26

8.1. DETAIL OF STUDY VISITS ... 26
8.1.1. Inclusion visit and randomization ... 26
8.1.2. Baseline visit at T0 ... 26
8.1.3. Phase 1 (T1-T3) ... 27
8.1.4. Wash out phase ... 27
8.1.5. Phase 2 (T4-T6) ... 27
8.1.6. Phase 3 at T7 ... 27
8.1.7. Study termination ... 27
8.2. LIST OF DATA DIRECTLY RECORDABLE ON THE CRF ... 27

9. PATIENT SELF MEASUREMENT TO BE RECORDED IN PATIENT DIARY ... 27

10. ASSIGNEMENT OF STUDY TREATMENT ... 27

10.1. RANDOMIZATION ... 27
10.2. TREATMENT ALLOCATION ... 28
10.3. BLINDING ... 28
10.4. EMERGENCY CODE ... 28

11. ADVERSE EVENTS AND SERIOUS ADVERSE EVENTS (FOR DRUGS) ... 28

12. ADVERSE EVENTS AND SERIOUS ADVERSE EVENTS (FOR MD) ... 28

12.1. DEFINITIONS (ISO 14155-2011) ... 28
12.1.1. Adverse events (AE) ... 28
12.1.2. Adverse Device Effect (ADE) ... 28
12.1.3. Serious Adverse Event (SAE) ... 28
12.1.4. Serious Adverse Device Effect (SADE) ... 29
12.1.5. Unanticipated Serious Device Effect (USADE) ... 29
12.1.6. AE of Special Interest ... 29
12.1.7. Device Deficiency ... 29
12.2. RECORDING OF AES/SAES ... 30
12.3. EVALUATION OF AES/SAES ... 30
12.3.1. Intensity ... 30
12.3.2. Causality ... 30
12.3.3. Occurrence ... 31
12.3.4. Expectedness ... 31
12.3.5. Seriousness ... 31
12.4. REPORTING OF AES / SAES / DDS AND FOLLOW-UP ... 32
12.5. SAFETY REPORT ... 33

13. ETHICS AND REGULATORY ISSUES ... 33

13.1. ETHICS COMMITTEE ... 33
13.2. COMPETENT AUTHORITY ... 33
13.3. AMENDMENTS ... 33
13.4. CONDITION FOR MODIFYING OR TERMINATING THE STUDY ... 34
13.5. PATIENT DATA PROTECTION ... 34
13.6. PATIENT'S INFORMATION AND INFORMED CONSENT ... 34
13.6.1. General process for obtaining informed consent ... 35

14. ARCHIVING OF STUDY DOCUMENTATION ... 35

15. INVESTIGATIONAL PROCEDURES ... 35

15.1. TRAINING ... 35
15.2. MONITORING ... 36
15.2.1. Deviations from clinical investigation plan ... 36

15.3. DIRECT ACCESS TO SOURCE DOCUMENTS ... 36
15.4. SOURCE DATA VERIFICATION ... 37
15.5. AUDITS AND INSPECTIONS ... 37

16. STUDY ORGANIZATION ... 37

16.1. INVESTIGATORS ... 37
16.1.1. Coordinating and Principal Investigators ... 37
16.2. STUDY ADMINISTRATION ... 37
16.2.1. Steering Committee ... 37
16.2.2. Data Safety Monitoring Board (DSMB) ... 37
16.3. OTHER STUDY CONTACTS ... 38
16.3.1. CRO ... 38

17. STATISTICAL METHODS ... 38

17.1. POPULATION SIZE ESTIMATION ... 38
17.1.1. Sample size calculation ... 38
17.2. CODING ... 38
17.3. STATISTICAL ANALYSIS ... 38
17.4. STATISTICAL ANALYSIS ... 38

18. DATA MANAGEMENT ... 40

18.1. CASE REPORT FORM ... 40
18.2. DATA ENTRY AND STORAGE ... 40
18.3. DATA MANAGEMENT AND QUALITY CONTROL ... 40

19. ADMINISTRATIVE PROCEDURES ... 41

19.1. STUDY DOCUMENTS AND RECORD KEEPING ... 41
19.2. CONFIDENTIALITY AND DATA PROTECTION ... 41
19.3. FINANCING AND INSURANCE ... 41
19.3.1. Fixed duty ... 41
19.3.2. Insurance ... 41
19.4. RESULT PROPERTY ... 42
19.4.1. Confidentiality ... 42
19.5. PUBLICATION OF STUDY RESULTS ... 42

20. REFERENCES ... 42

21. LIST OF APPENDICES ... 42

LIST OF AMENDMENTS

"NOT APPLICABLE"

Page / Section	Version no. 1.0 of 2013 11 04	*New* Version no. 2.0 of yyyy mm dd	Subst. Modif.?
	(1) .	(2) .	(3)

(1) Please, fill in with the original text
(2) Please, fill in with the modified text: updated text should preferably be mentioned in ***Bold Italic*** or highlighted.
(3) Rationale for modification is mandatory. If justification of substantial modifications is already mentioned in a document (for e.g. into the Application Letter for Substantial modification), please indicate the name of document in which it can be found.

LIST OF ABBREVIATIONS

ADE	Adverse Device Effect
AE	Adverse Event
AGE	Advanced Glycation Endproducts
β2-m	β2-microglobulin
BfArM	Bundesinstitut für Arzneimittel und Medizinprodukte
CRF	Case Report Form
CRO	Clinical Research Organization
GCP	Good Clinical Practices
CI	Chronic inflammation
CKD	Chronic Kidney Disease
CRF	Case Report Form
CRO	Clinical Research Organization
CRP	C-reactive Protein
CVC	Central venous catheter
GCP	Good Clinical Practices
CA	Competent Authority
ESRD	End stage renal disease
HD	Hemodialysis
Hb	Hemoglobin
HCO	High cut-off
HF	High Flux
IMP	Investigational Medical Product
IB	Investigator's brochure
IFU	Instruction for use
Kt/V	*K* (clearance) multiplied by *t* (time)
Min	Minute
Ml	Milliliter
mRNA	Messenger ribonucleic acid
MCO	Medium cut-off
PVD	peripheral vascular disease
P210H	Polyflux 210 H
PBMC	Peripheral blood mononuclear cells
Q_B	Blood flow rate
Q_D	Dialysis flow rate
Q_{UF}	Ultratfiltration flow rate
RBV	Residual blood volume
SD	Standard deviation
SEM	Standard deviation of the mean
SmPC	Summary of product characteristics
SAE	Serious Adverse Event
TMP	Transmembrane pressure

Note: for the purpose of this document:

- "Clinical Study Protocol" is similar to "Clinical Investigation Plan" or "Protocol",

"Control Product" is similar to "Comparator Product".

SYNOPSIS

Title	*Permeability Enhancement to Reduce Chronic Inflammation _ Medium cut off (MCO) (Study no 1502)*
Phase	Pilot Study
Steering Committee	Prof. Schindler, Prof. Girndt, Dr. Storr
Data Safety Monitoring Board	No Data Safety Monitoring Board
Coordinating Investigator	PROF DR. MED. MATTHIAS GIRNDT , GERMANY
Sample size	50 randomized patients (25 patients/ randomization group)
Target population and indication	ESRD, Hemodialysis (HD)
Investigational product	MCO-Ci 400 (MCO)
Comparator product	Revaclear 400 (HF)
Treatment groups	2 treatment groups (MCO vs HF) in cross over design
Study treatment duration	6 months
Dosage	Hemodialysis, 3 times weekly
Concomitant medication	Any medication or treatment required for the welfare and health of the patients enrolled in the study will be administered at the investigator's discretion. All such concomitant medication shall be reported on the CRF and this, as soon as the signature of the Patient Consent Form and up the end of the study for the concerned patient.
Planned no. of Centers / Countries	2 sites / one country (Germany)
Provisional subject schedule	- screening/ inclusion : 2 weeks<br>- Run-in period, 1 month<br>- Phase 1, 1 month<br>- Wash out phase, 1 month<br>- Phase 2 , 1month<br>- Phase 3, 2 months<br>Total study duration (per subject): 6 months
Recruitment period	2 weeks
Planned First Patient In (FPI)	February 2014

Planned Last Patient Out (LPO)	July 2014
Study rationale	MCO eliminates pro inflammatory molecules which down-regulates chronic inflammation in ESRD pts
Study design	Bi-centric, controlled, randomized, open-label, cross-over study
**Objectives**	
Primary objective	Reduction of inflammation
Secondary objective	Elimination of inflammatory molecules and uremic toxins from patients blood, Safety, exploratory investigations
**Assessment parameters**	
Parameters for primary objective	TNF-α mRNA in PBMC
Parameters for secondary objectives	Safety events, routine laboratory, mRNA of inflammatory mediators, elimination kinetics of proteins (plasma levels), experimental ex vivo analysis
**Endpoints**	
Endpoint of primary objective	Significant lower pre dialytic TNF-α mRNA expression level MCO versus HF after 4 weeks treatment time (T1/T3 versus T4/T6)
Endpoint of secondary objective	- safety events (SAE, AE, DD, other observations)<br><br>- routine laboratory diagnostic parameter<br><br>- IL-6 mRNA expression level in PBMC after 4 weeks (T1/T4 versus T3/T6) and 3 month treatment (T7)<br><br>- TNF-α mRNA expression level in PBMC after 3 month treatment (T7)<br><br>- Kinetics of selected uremic toxins during one dialysis session (with a subset of 10 patients only at the study site Halle)<br><br>- Change of plasma proteins (explorative markers) after 4 weeks (T1/T3 versus T4/T6) and 3 month treatment (T7).<br><br>- Experimental ex vivo analysis of patient plasma and dialysis fluid (proteomics, effects on cellular assays, AGEs)<br><br>- Change of µRNA (explorative markers) after 4 weeks (T1/T3 versus T4/T6) and 3 month treatment (T7).

Selection criteria	
Inclusion criteria	- CKD5 (GFR < 15ml/min/ 1.73m²)<br>- Dialysis treatment for ≥ 3 months<br>- Dialysis 3x weekly<br>- Vascular access by fistula or CVC<br>- Age > 18 and < 99 Years<br>- Ability to give written informed consent
Exclusion criteria	- Missing informed consent form<br>- current clinically manifested infection or within the last two weeks<br>- current CRP-value > 50mg/L or within the last two weeks<br>- Intake of any medication applied for immunosuppressive purposes<br>- Pregnancy or lactation<br>- Participation in a different interventional study
Safety (AE, SAE, DD)	All AE, SAE and other safety issues will be collected and described as soon as start of run in phase up to the end of the study for the concerned patient.
Data Quality Assurance	Monitoring procedures defined by the sponsor will be followed, in order to comply with ICH-GCP guideline. Each site will be visited at regular intervals by a monitor to ensure compliance with the protocol.<br><br>The study management software eResearchNetwork, commercially available validated software approved by the Food and Drug Administration (FDA), will be used for data capture and query management.<br><br>Data entry and "second look" will be carried out by two independent persons. Data will be evaluated for consistency, accuracy and completeness regularly. After completion of data capture data base will be closed and the data will be transferred into the statistic software (SAS). SAS data files will be transferred to the trial statistician.<br><br>Database setup, validation, data management and database lock will be performed by the KKS Halle according to the appropriate SOPs.
Statistical analysis	Primary: Baseline adjusted analysis of intraindividual treatment effect in crossover design (TNF-α mRNA expression level: delta (T1/T3 resp. T4/T6) MCO minus delta HF) using Generalized Estimating Equations. Test for superiority. Intent to Treat analysis in modified ITT population to be defined in a blind data review. No interim analysis. Secondary analysis: in ITT and PP population, secondary endpoints identical statistical methods. Exploratory analysis of phase 3 Safety analysis for all subjects receiving at least one intervention (safety population)<br>.
Regulatory	The study will be submitted to Ethics Committee and notified to Competent Authorities before study start and patient enrollment.<br><br>The study will be conducted in accordance with current local regulations and international applicable regulatory requirements (ISO 14155-2011, European Directive 2007/47/EC, European Directive 95/46/EC relating to data protection and the Declaration of Helsinki, last amendment at 64th WMA General Assembly, Fortaleza, Brazil, October 2013 (www.wma.net)).

STUDY SCHEDULE

T = Time point for blood sampling		T0	T1	T2	T3	T4	T5	T6	T7	Each week
HD session	-18 to -12	-12	pre 1	post 1	pre 13	Pre 25	Post 25	Pre 37	Pre 61	
Visit	1 (screening, inclusion)	2 (baseline)	3	3	4	5	5	6	7	
Demographic Medical history	X									
Inclusion / Exclusion Criteria	X									
Informed Consent	X									
Physical Examination	X								X	
Concomitant Therapy	X	X	X		X	X		X	X	
Treatment Allocation		X	X			X		X		
AE / SAE										X
Primary endpoint			X		X	X		X		
Routine lab		X	X		X	X		X	X	
Study specific lab			X	X	X	X	X	X	X	
Kinetics[1]			X	X		X	X			
Study Termination Form									X	

[1] Only to be done in 10 patients at study site Halle

1. INTRODUCTION

1.1. BACKGROUND AND RATIONALE

About 1.7 million people worldwide rely on hemodialysis due to endstage renal disease (ESRD). Although progress has been made in therapy in the dialysis treatment for the past years, dialysis patients still have a significantly reduced life expectancy compared to people with normal kidney function at the same age. In particular, the mortality due to cardiovascular diseases is 10- to 100fold higher than for healthy people (Weiner, 2004). Today, it is widely accepted that chronic inflammation which is pronounced with about one third of all dialysis patients, can cause cardiovascular complications via different pathophysiologic mechanisms (Vanholder, 2005).

A common factor in many of the potential causes of chronic inflammation is the presence of larger molecules in the blood circulation, either through retention and accumulation due to lack of renal elimination and insufficient clearance by the dialysis treatment (uremic toxins) or through enhanced generation by (continuously or repeatedly) on-going inflammatory processes (acute phase response) or through both. Elimination of such

molecules is limited by the permeability of the dialysis membranes, in addition to procedural limitations regarding duration and frequency of the dialysis treatment. Although the elimination of certain larger molecules (e.g. ß2-microglobulin) can be increased by applying convective treatment modes (HDF/HF) and despite recent advances in studying more frequent and/or prolonged hemodialysis treatments the principal limitation regarding effective elimination of a broad range of large molecules can only be partially overcome by this, when relying on membranes with conventional molecular cut-off permeability.

The medium cut-off dialysis membrane has been developed by Gambro to provide a significantly extended molecular cut-off compared to conventional high-flux membranes. The medium cut-off membrane allows for a high permeability of molecules up to a molecular weight of 45 kDa and has a still limited permeability for albumin (68 kDa), which is lower as it is found in high cut-off dialyzers (HCO1100, 1.1 m² membrane surface area, and Theralite, 2.1 m² membrane surface area, from Gambro). The medium cut-off membrane is contained in the CE-marked study product MCO-Ci 400 dialyzer (1.8 m² membrane surface area) available from Gambro.

The high molecular permeability of the high cut-off membrane in HCO1100 and Theralite allows for significant clearance of cytokines and other pro-inflammatory solutes by hemodialysis. This has been demonstrated for IL-8 (10 kDa), $beta_2$-microglobulin (12 kDa), Leptin (16 kDa), IL-1beta (17 kDa), IL-1ra (17 kDa), complement factor D (24 kDa), IL-6 (26 kDa), kappa-FLC (23 kDa), lambda-FLC (45 kDa) and TNF-alpha (51 kDa) in ex vivo studies (Uschino *et al.* ASAIO J 2002, Lee *et al.* Int J Artif Org 2003, Lee *et al.* Int J Artif Org 2004) and in clinical investigations in different patient populations (acute renal failure after multiple myeloma: Hutchison et al JASN 2007, Hutchison et al Artif Org 2008; Heyne *et al.* NDT 2007; acute renal failure after sepsis/septic shock: Morgera et al AJKD 2004; Haase *et al.* AJKD 2007; chronic renal failure: Schindler *et al.* Blood Purif 2002). As the medium cut-off membrane allows a similar clearance of middle molecules as the high cut-off membranes, a comparable effect of the new membrane can be expected.

In two recent pilot clinical trials two to three weeks hemodialysis treatment of chronic HD patients with the HCO1100 dialyzer has been shown to significantly down-regulate monocyte cell surface proteins: CCR2, CX3CR1, CD11b and CD163 (Hutchison *et al.* JASN 2008); CD 162, CD 11 and CD 181 (Zickler *et al.* JASN 2009). A lower increase in serum levels of pro-inflammatory cytokines (IL-1b, IL-6, TNF-alpha) during HD sessions compared to treatment with high-flux dialyzers has been observed in addition to a significant reduction of pre-dialysis serum free kappa and lambda light chain concentrations (Hutchison *et al.* JASN 2008) as well as a trend for improvement of aortic stiffness (pulse wave velocity, Zickler *et al.* JASN 2009). In a further pilot trial, hemodialysis treatment with Theralite for every second treatment over a period of 12 weeks led to a significant rise in pre-treatment hemoglobin levels, a significant reduction of pre-treatment hepcidin values, and a non-significant reduction of ESA dose (Longhena *et al.* NDT 2013).

The main goal of this project is the evaluation of the new, highly porous and selective dialysis membrane (MCO-Ci 400) for the treatment of patients with ESRD in hemodialysis mode and to study its potential to improve chronic inflammation.

The hemodialyser MCO-Ci 400 is a CE marked medical device (class IIb) and the intended use will be followed in this study as described in the IFU. The clinical evaluation of the study product MCO-Ci 400 was carried out according to the requirements of the European Directive 2007/47/EEC based on pre-clinical testing data of MCO-Ci 400 and on clinical data from published literature of equivalent hemodialysers (HCO1100, Theralite and Revaclear 400).

In this planned study the MCO-Ci 400 will be the first time used in a clinical setting. The clinical experiences with the equivalent hemodialysers (HCO1100, Theralite and Revaclear 400) have demonstrated the safe use in the ESRD patient population. Information is provided in the investigator brochure (IB).

With this study, the performance the MCO-Ci 400 dialyser shall be investigated in comparison to the application of the standard highflux membrane (Revaclear 400).

Endpoints of this investigation are changes of cellular inflammatory markers (e.g. TNF-α mRNA expression level in PBMC) induced by the application of the MCO membrane (MCO-Ci 400) within short (4 weeks) and long term (3 month) treatment periods.

The primary endpoint TNF-α mRNA expression has been selected based on the results of a previous clinical investigation with a HCO1100 dialyser (Perci Study, unpublished).

A kinetic modelling of selected inflammatory markers and uremic toxins potentially removed from the blood by the MCO membrane (MCO-Ci 400) will provide further information on the clinical performance (mode of action) of the MCO membrane (MCO-Ci 400). In addition this kinetic modelling will also provide data for the estimation of the distribution volume of these inflammatory markers and uremic toxins, which may have impact on treatment modalities (e.g. optimised duration of dialysis sessions, frequency of dialysis sessions).

Further, quantitative analysis of specific protein patterns from patient plasma and dialysis fluids shall be done (proteomics). An additional innovative approach is the determination of samples effects in ex-vivo assays.

Beside the evaluation of the performance this study will also investigate safety by collecting information on the occurrence of safety related events.

The chosen cross over study design is suitable to verify primary and secondary endpoints with a lower patient number as compared to a parallel group study design.

1.2. Benefits and risks for the study population

a) Anticipated clinical benefits are the efficient removal of uremic toxins. Further MCO-Ci 400 may reduce the micro-inflammation.

b) Anticipated adverse device effects when used according to the Instruction for Use (IFU) are not known.

c). Foreseeable residual risks for the patients included in this clinical investigation do not differ from those usually observed during this type of treatment.

d) Risks associated with participation in the current clinical investigation are related to the volume of blood drawing (493 mL). No additional punctures of vessels are required compared to routine dialysis treatments.

e) Risk assessment results related to the investigational product are reported in the Investigator's Brochure.

f) Steps that will be taken to control or mitigate the risks: During the study all patients are seen thrice weekly by a physician.

g) There is a positive Risk-to-benefit ratio, when comparing foreseen increased risks of MCO-Ci 400 versus potentially improved removal of uremic toxins/ inflammatory mediators.

1.3. Justification of treatment periods and evaluation schedule

Since the MCO membrane has a nearly similar removal capacity for molecules up to 45 kDa as the HCO membrane we justified the treatment period based on the experiences of clinical studies with HCO1100. In the PERCI study (unpublished data) we detected mRNA levels of TNF-α and IL-6 to be significantly reduced after 3 weeks treatment period. Among the soluble inflammation mediators, some were reduced by 3 weeks of treatment, some were not. However the reduction of serum levels of several soluble inflammation mediators was

more pronounced after three weeks of treatment compared to two weeks. We therefore have chosen a 4 weeks treatment period for the primary endpoint TNF-α mRNA for this study.

Since the reduction of pre-dialytic values of soluble inflammatory molecules (secondary endpoints) is expected to take longer, a 12 weeks prolonged treatment period has been chosen to follow pre dialytic values of inflammatory mediators.

Evaluation schedule:

-2 weeks screening/ recruiting

1.) Run in Phase: 4 weeks (HF)
T 0 (Baseline)
2.) Phase 1: 4 weeks (HF/ MCO)
T1 –T3
3.) Wash out: 4 weeks (HF)
4.) Phase 2: 4 weeks (HF/ MCO)
T4-T6
5.) Phase 3: 8 weeks (HF/ MCO)
T7

2. STUDY OBJECTIVE

2.1. PRIMARY OBJECTIVE

The objective of this clinical investigation is the reduction of inflammation in chronic hemodialysis patients as detected by mRNA transcript rates in circulating peripheral blood mononuclear cells.

2.2. SECONDARY OBJECTIVES

Elimination of inflammatory molecules and uremic toxins from patient's blood
Evaluation of the occurrence of safety related events.

2.3 Exploratory analysis

Phase 3 of the study will be analysed using exploratory statistical methods as defined in section statistical analysis.

3. STUDY PARAMETERS AND ENDPOINTS

3.1. PRIMARY ENDPOINTS

Significant lower pre dialytic TNF-α mRNA expression level MCO versus HF after 4 weeks treatment time (T1/T3 versus T4/T6)

3.2. SECONDARY ENDPOINTS

- Safety events (SAE, AE, DD, other observations)

- Routine laboratory diagnostic parameter

- IL-6 mRNA expression level in PBMC after 4 weeks (T1/T3 versus T4/T6) and 3 month treatment (T7)

- TNF-α mRNA expression level in PBMC after 3 month treatment (T7)

- Kinetics of selected uremic toxins during one dialysis session (with a subset of 10 patients only at the study site Halle)

- Change of plasma proteins (explorative markers) after 4 weeks (T1/T3 versus T4/T6) and 3 month treatment (T7).

- Experimental ex vivo analysis of patient plasma and dialysis fluid
 (proteomics, effects on cellular assays, AGEs)

- Change of µRNA (explorative markers) after 4 weeks (T1/T3 versus T4/T6) and 3 month treatment (T7).

4. STUDY DESIGN

The clinical study design is the following:

- Bi-centric
- Open label
- Controlled, cross over
- randomized during run-in period
- pilot

Studien Design Perci-MCO Study No. 1502

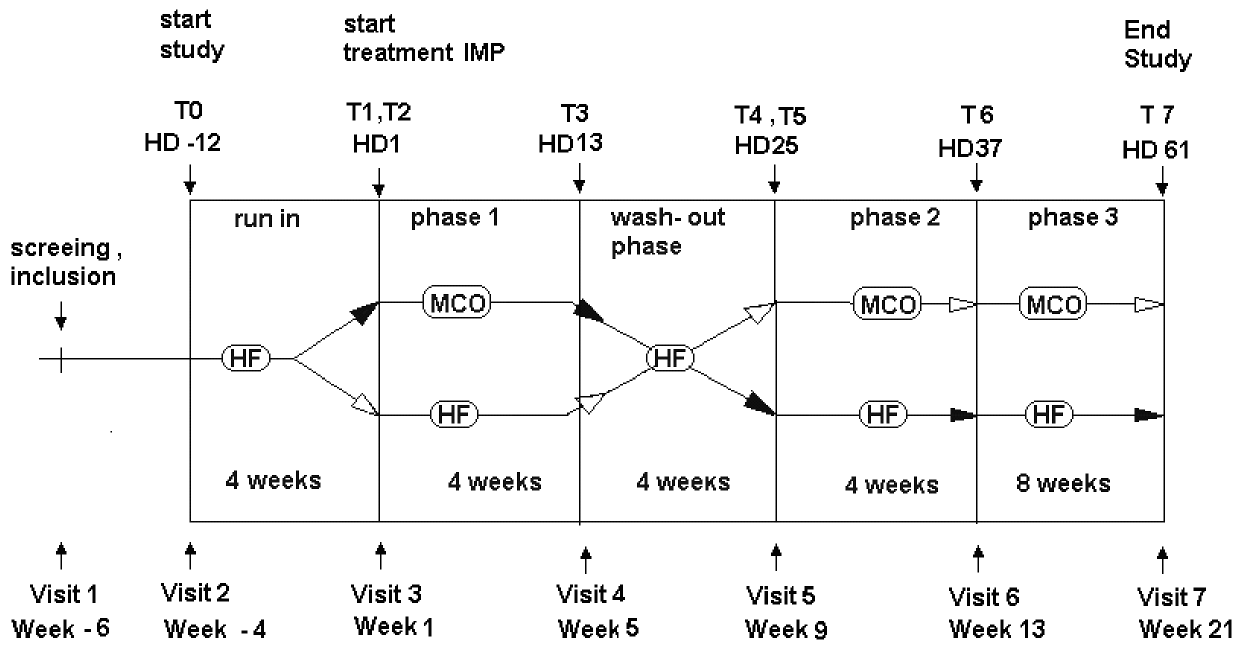

4.1. STUDY DURATION

The study is planned to start in February 2014 (i.e. First Patient In) and to stop in July 2014 (Last patient Out). The recruitment is planned for 2 weeks until February 15th.

The dates of the study duration are defined as follows:

- Date of clinical investigation initiation = First Patient In = date of the first patient starting first HD treatment of "run in phase" (= T0 = Baseline = visit 2)

- Completion date of the clinical investigation = Last patient Out = date of the last visit (= visit 7) of the last patient undergoing the study.

4.2. STUDY PERIODS

The study will be divided in five steps as describes in the figure below:

- Run in phase
- Phase 1
- Wash out phase
- Phase 2
- Phase 3

Note that Phase 3 is not part of the cross over design.

4.3. STUDY SUSPENSION AND DISCONTINUATION RULES

The study should be suspended if suspicion of an unacceptable risk to subjects arises during the clinical investigation, or when so instructed by the EC or regulatory authorities. Risk to subjects could be indicated when any of the following events occurs:

- Device malfunction, considered by the investigators and/or Gambro as possibly leading to a deficiency in the patient's treatment,
- Repeated patient discomfort with the product,
- Unexpected/Unanticipated adverse event/effect.

The sponsor shall suspend the clinical investigation while the risk is assessed. The sponsor shall terminate the clinical investigation if an unacceptable risk is confirmed.
Decision to restart the study will be made jointly by Gambro and the Steering Committee following evaluation of the problems encountered.

4.3.1. Procedure for resuming the clinical investigation after temporary suspension

When the sponsor concludes an analysis of the reason(s) for the suspension, implements the necessary corrective actions, and decides to lift the temporary suspension, the sponsor shall inform the principal investigators, the ECs, and, where appropriate, the regulatory authority of the rationale and provide them with the relevant data supporting this decision.

Note: The usual lines of communication are sponsor <=> principal investigator or sponsor <=> EC, and sponsor <=> regulatory authority.

Concurrence shall be obtained from the ECs and, where appropriate, regulatory authorities before the clinical investigation resumes.
If subjects have been informed of the suspension, the principal investigator or authorized designee shall inform them of the reasons for resumption.

4.4. PRODUCT AND MATERIAL

All the products required during this clinical investigation will be provided free of charge by the sponsor (i.e. investigational product and comparator product).

The CE-marked MCO-Ci 400 dialyser can be used for the removal of plasma components with molecular weights up to 45kDa and the dialyser has to be applied according to the IFU and the study protocol.
The material composition of the study membrane can be looked up in the table “Table 1 Raw materials” .

Raw materials (in finished product to customer)

[redacted]

Table 1: Components and Materials of the membrane

<u>Claims and intended performance :</u>

- The investigational product (MCO) is intended for blood purification (elimination of uremic toxins and inflammatory mediators) in chronic dialysis patients with ESRD. It is expected that the elimination of uremic toxins and inflammatory mediators with MCO reduces the inflammatory status.
- The comparator Revaclear will be used 4 weeks in the “run in phase and 4 weeks in the wash out phase
- MCO Ci-400 and Revaclear 400 will be sent by Gambro R&D directly to the study sites (KfH) in Berlin (Investigator Dr. Zickler) and Halle (Investigator Dr. Fiedler).

4.4.1. General description of Investigational Product

For a general description of the investigational product MCO please see the IFU. For a more detailed description of the investigational product MCO including summaries of literature data on safety and biocompatibility as well as safety and risk evaluations please see the *Investigator* Brochure.

4.4.1.1. Manufacturing process

This product is CE marked and manufactured according to the essentials requirements described in the European Directive 2007/47/EEC. It belongs to Class IIb device according to 93/42/EEC annex IX.
The study product is packaged with a standard Gambro package and sent to the investigator for the clinical investigation. All manufacturing and packaging steps are performed at Gambro Dialysatoren GmbH, Hechingen, which is certified for medical device production.

Summarized information related to manufacturing of the investigational product is available in the Investigator's brochure.

4.4.1.2. Investigational Product labeling

Each investigational device will bear the following information:

- Identification label bearing all mandatory and regulatory information, including sterilization and shelf life
- Barcode label for product manufacturing traceability purposes.

Orange label bearing the mention "For investigational purpose only" will be stuck on each box.

4.4.2. General description of Comparator Product

- The comparator Revaclear used in this study is intended for blood purification in chronic dialysis patients with ESRD.
- The comparator Revaclear 400 (HF) is a state of the art, standard high flux hemodialyser for blood purification in chronic dialysis patients with ESRD.

For a general description of the comparator Revaclear 400 (HF) please see the IFU.

4.4.2.1. Manufacturing process

This product is CE marked and manufactured according to the essentials requirements described in the European Directive 2007/47/EEC. It belongs to Class IIb device according to 93/42/EEC annex IX.

4.4.2.2. Comparator Product labeling

Each investigational device will bear the following information:

- Identification label bearing all mandatory and regulatory information, including sterilization and shelf life
- Barcode label for product manufacturing traceability purposes.

Orange label bearing the mention "For investigational purpose only" will be stuck on each box.

4.4.3. Other material

The clinical setup of equipment to be used in the clinical study is the same setup that is used in regular hemodialysis (HD) treatments. The investigational and comparator products will be connected to a dialysis monitor.

The dialysis machine controls blood flow rate, dialysis fluid flow rate, ultrafiltration rate, dialysis fluid composition, temperature and monitor pressures (arterial, venous and transmembrane pressure).

The following equipment is needed at each site to be used during the HD treatments, all devices being CE-marked:

- Standard dialysis monitor
- Standard arterial and venous blood lines

4.5. PRODUCT ACCOUNTABILITY

The investigators at each site will be supplied with the products to be investigated during this clinical study. The accountability document must be filled in by the person in charge of receiving the products at site. The recording method defined and approved during the "study initiation visit" shall apply and the document ("GCP forms") shall be filled out as and when required.

The investigational and comparator products intended for the clinical study must be stored in a safe place with restricted access and used to treat the patients included in the study under the sole supervision of the investigator.
Both the principal investigators (or delegated person) and Gambro Study Monitors are jointly responsible for recording both the products supplied as well as the number of products used during the study, in addition to material.
The principal investigator is solely responsible for keeping records of updated documents according to the following requirements:

a) The date of receipt at the study site,
b) Identification of each investigational and comparator device (batch number/serial number or unique code),
c) The expiry date, if applicable,
d) The date or dates of use,
e) Subject identification,
f) Date on which the investigational and comparator device was returned/explanted from subject, if applicable, and
g) The date of return of unused, expired or malfunctioning investigational and comparator devices, if applicable.

At each visit, the Study Monitor must ensure that the investigator has sufficient number of products (or material) at his/her disposition, that products have been duly counted, and that the products are being used according to their specifications.

5. SELECTION AND WITHDRAWALS OF PATIENTS

5.1. STUDY-SPECIFIC DEFINITIONS

Other observations which do not fall under the definition of AE, SAE or Device Deficiency, concern information related to any Gambro product (excepted the investigational product or the potential comparator) that could indicate a safety risk for the patient. This could include deficiencies related to the identity, quality, durability, reliability, safety, effectiveness, or performance of a Gambro product, such as error in labeling, packaging, misuses, spontaneous report by a healthcare professional, etc.

5.2. STUDY POPULATION

The patients to be included in this clinical study are suffering from chronic renal failure and are usually treated by standard high-flux hemodialysis. These patients shall meet the inclusion and exclusion criteria defined for this study.

5.2.1. Vulnerable population

The current clinical study will not involve a vulnerable population.

5.3. INCLUSION CRITERIA

- CKD5 (GFR < 15ml/min/ 1.73m^2)
- Dialysis treatment for ≥ 3 months

- Dialysis 3x weekly
- Vascular access by fistula or CVC
- Age > 18 and < 99 Years
- Ability to give written informed consent

5.4. - EXCLUSION CRITERIA

Exclusion criteria are the following:
- Missing informed consent form
- Current clinically manifested infection or within the last two weeks
- Current CRP-value > 50mg/L or within the last two weeks
- Intake of any medication applied for immunosuppressive purposes
- Pregnancy or lactation
- Participation in a different interventional study

5.5. RECRUITMENT AND SAMPLE SIZE

Recruitment for the study will start after Ethics Committee and competent authorities' approval. After checking inclusion and exclusion criteria, the patient will receive the patient information form. Patients will be considered enrolled in the study when the informed consent has been signed.

During a previously conducted clinical study (Perci study, unpublished) with ESRD patients we experienced a 20% drop out rate by a study length of four months. Due to the prolonged study length of total 6 months, we expect a drop out rate of 30% for this study.

Therefore a total of 50 patients, including a 30% drop out rate, are to be included in the study.

- Ø 25 patients will be randomized to the treatment group starting with MCO treatment
- Ø 25 patients will be randomized to the treatment group starting with HF treatment

5.6 PATIENT WITHDRAWAL AND REPLACEMENT

Patients are free to withdraw from the study at any time without having to justify their decision. When a patient decides to withdraw from a study, he/she will be systematically contacted and given the opportunity to provide information about the reason(s) of his/her withdrawal and possible occurrence of an adverse event.

If at anytime the investigator considers that malfunctions/AEs could possibly lead to a deficiency in the patient's treatment, the treatment in question shall be discontinued and replaced by a standard treatment.

Patients may be withdrawn from the study at any time and at the investigator's discretion.

Patients must be withdrawn from the study if any of the following occurs:

- Any event which, in the opinion of the investigator, may either endanger the health of the patient, or may incorrectly influence the result of the study
- Patients refusing or not considered capable, as judged by the investigator, of following instructions of the study.

If a patient be withdrawn from the study, the Investigator has to inform the Study Manager in the shortest possible time, irrespective of the reason for withdrawal. Reason for withdrawal should be recorded in the Case Report Form (CRF).

If withdrawal is related to the possible occurrence of adverse events, the patient will be followed up according to the adverse event procedure.

Withdrawn patients will not be replaced as a drop out rate of 30% is included in the patient size evaluation.

6. PATIENT TREATMENT

6.1. Recommendation for study products implementation

The procedure for product implementation is described in the Instructions For Use, available in the Investigator's Brochure (IB). All recommendations and warnings mentioned in these instructions shall be strictly taken into account.

Anytime the investigator considers that reported AEs could possibly lead to a deficiency in the patient's treatment.

6.2. Concomitant medication

Any medication or treatment required for the welfare and health of the patients enrolled in the study will be administered at the investigator's discretion. All such concomitant medication shall be reported on the CRF and this, as soon as start of run in phase up to study end.

6.2.1. Medication allowed

Any medication commonly prescribed for ESRD patients

6.2.2. Medication not allowed

Any medication applied for immunosuppressive purposes.

6.3. Treatment details

6.3.1. Dialysis treatment

Standard HD care three times per week

6.3.2. Heparin strategy

The type of anticoagulation, the dosage, and the administration during HD sessions must be continued as individually prescribed during the study period and must be noted in the CRF.

6.4. Patient medical care after clinical study closure

After the clinical investigation has been completed patients continuing renal replacement therapy and general medical care will be provided for the subjects

7. LABORATORY AND ASSESSMENT

Blood pressure will be determined according to routine procedure.

Blood sampling will be done as described in the sampling schedule. Central Laboratory analyses are summarized in Table 2 in the section Central analyzes. Further blood samples are drawn for exploratory analysis:

- Explorative Proteomic analysis at NMI Reutlingen, Germany
- Explorative analysis of AGE´s at Food Chemistry Laboratory Halle, Germany
- Explorative analysis at Charité Berlin, Germany
- Explorative analysis at University Clinic Halle, Germany
- Explorative analysis at Gambro laboratories Hechingen, Germany
- Explorative analysis of µRNA at Internal Medicine Department of Homburg University, Germany

The labelling of laboratory samples must ensure that the laboratory assessments are executed in a blinded fashion by the laboratory personal.

7.1. STUDY VISITS

Seven Study visits will be done (Visit 1-7)

- Visit 1 Screening and Inclusion visit (within two weeks prior start of “run in phase”)
- Visit 2 (start “run in phase”): week 1; dialysis session -12 (week -4)
- Visit 3 (start Phase 1,week 1; dialysis session 1):
- Visit 4 (start Wash-out period: week 5; dialysis session 13)
- Visit 5 (start Phase 2: week 9; dialysis session 25)
- Visit 6 (start phase 3, week 13;dialysis session 37)
- Visit 7 (end Phase 3:week 21; dialysis session 61)

7.2. SAMPLING SCHEDULE

There are three efficacy and safety periods, each period lasting at the most for eight weeks. During each week three HD treatments shall be performed.

7.3. SAMPLING DETAILS

<u>8 sampling time points</u>
T0 pre dialysis session HD -12
T1 pre dialysis session HD 1
T2 post dialysis session HD1
T3 pre dialysis session HD13
T4 pre dialysis session HD 25
T5 post dialysis session HD25
T6 pre dialysis session HD 37
T7 pre dialysis session HD 61

- Kinetics: 10 patients x 2 time points / à 6mL = 120mL

In 10 selected patients kinetic monitoring of inflammatory proteins will be done at 2 time points in the study (first treatment with MCO, first treatment with Revaclear). For this monitoring, an additional total of 6ml blood per time point will be drawn.

- Routine lab: 6 sampling à 5mL =30mL
- 7 sampling exploratory lab à 15mL = 105ml (Charité)
- 7 sampling exploratory lab à 10mL = 70ml à 14,2 / 5,5mL =82ml (Halle)
- 7 sampling exploratory lab à 5mL = 35ml (Halle Lebensmittelchemie)
- 7 sampling exploratory lab à 15mL = 105ml (Gambro)
- 7 sampling exploratory lab à 3mL = 21ml (NMI)
- 7 sampling exploratory lab à 1mL = 7 ml (Homburg University)

---- -- ----
Total blood volume / per patient 493 mL

- <u>Blood samples</u>: No additional puncturing of the patient is necessary as the sampling will be performed from the extracorporeal circuit’s arterial access needle.

In total a maximum of 493 mL blood volume will be collected during the entire study (6 month).

7.4. Parameters and laboratory analyzes

In order to prevent any variation between tests, guidelines will be followed.

7.4.1. Local analyzes

Locally analyzed samples will be performed according to local routine. Methods, units and ranges for the primary and secondary endpoint parameters shall be available to sponsor before study start.

7.4.2. Central analyzes

Parameters and the methodology for centrally analyzed study parameters are presented in *Table 2.* Sampling, handling and storage details will be describe in more details during training at study site.

Table 2

Centrally analyzed parameters *- by M. Girndt lab (Halle)*				
Parameter / Method	Quantitative rt-PCR	FACS		
mRNA TNFa	x			
mRNA IL-6	x			
mRNA sTNFR1	x			
mRNA sTNFR2	x			
mRNA sVCAM	x			
mRNA sIL-2R	x			
mRNA Adiponectin	x			
Monocyte CD14/CD16 subpopulations		x		
M-DC8 positive monocytes		x		
Centrally analyzed parameters *- by Gambro Hechingen (M. Hulko)*				
Parameter / Method	Elisa	HPLC	Nephelometric Assay	Fluorometric Assay
Oxidized Albumin		x		
Albumin-gebundene Toxine		x		
Beta 2-microglobulin			x	
Myoglobin			x	
Free Light Chain, kappa (κ-FLC)			x	
Free Light Chain, lambda (λ-FLC)			x	
a1-Microglobulin			x	
suPAR	x			
beta Trace protein			x	
Pentraxin 3 (long) (PTX-3)	x			
Complement Factor D	x			
Glutathion oxidized*	x			
GSH/ GSSG Ratio *				x
Fetuin A*	x			
MGP*	x			
Prolactin*	x			
YKL-40*	x			
Locally analyzed parameters in Charité and Halle				
Routine laboratory Charite				
Routine laboratory Halle				
Locally analyzed parameters at NMI				

Parameter	Plex 1	Plex 2	Plex 3	Plex 4
ESelectin	X			
IL6sR	X			
MIF	X			
PLA2G7	X			
Rantes	X			
sFas	X			
sgp130	X			
sICAM	X			
sRAGE	X			
sTNFR1	X			
sTNFR2	X			
sVCAM	X			
GMCSF		X		
GRO		X		
IFNg		X		
IL10		X		
IL12p40		X		
IL12p70		X		
IL17A		X		
IL1b		X		
IL1ra		X		
IL2		X		
IL4		X		
IL6		X		
IL8		X		
MCP1		X		
MIP1a		X		
MIP1b		X		
sCD40L		X		
sIL2Ra		X		
TNFa		X		
Adiponectin			X	
FGF23				X
Insulin				X
Leptin				X
Osteopontin**				X
Osteocalcin**				X
** Assay under evaluation				

EVALUATED PARAMETERS AND METHOD

- Sampling and handling of blood for primary endpoint analysis is detailed described in a SOP
- Methodology and calculation of mRNA expression analysis is detailed described in a SOP

8. PATIENT PARAMETERS TO BE RECORDED ON CRF

Primary endpoints: TNF-alpha mRNA

Secondary endpoints: Safety related events (AE, SAE, DD, and other events)
Long term mRNA expression of TNF-alpha and IL-6

The CRF is designed to record all patients' data (e.g. patient demography, laboratory data, concomitant medication, AEs, SAEs, etc.).

Each CRF is divided in the following parts:

Ø Inclusion visit and randomization	visit 1 (between HD -12 and HD -18)
Ø Baseline/ Run in phase:	visit 2 (week -4; dialysis session -12 ;T0)
Ø Phase 1:	visit 3 (week 1; dialysis session 1; T1,T2)
Ø Wash-out period:	visit 4 (week 5; dialysis session 13; T3)
Ø Phase 2:	visit 5 (week 9; dialysis session 25; T4,T5)
	visit 6 (week 13;dialysis session 37;T6)
Ø Phase 3:	visit 7 (week 21; dialysis session 61; T7)

- Ø Concomitant medications
- Ø Recording of AE medication
- Ø Safety event forms (AE, SAE, DD)
- Ø Study termination

8.1. DETAIL OF STUDY VISITS

8.1.1. Inclusion visit and randomization

A form summarizing the medical history of each patient (Patient presentation), shall be filled out and contain at least the following information:
- Patient's age and gender,
- Diagnosis and medical history
- Number of years in dialysis,
- Dialysis mode
- Prescription before entering clinical study
- CRP

8.1.2. Baseline visit at T0

A form summarizing the Run-In period shall be filled out and contain at least the following information: Biological parameters (hematology, biochemistry), usually checked at regular intervals on chronically hemodialyzed patients, will be reported on the same CRF.
- Prescription before entering clinical study
- Routine laboratory results

8.1.3. Phase 1 (T1-T3)

Required information:
Routine laboratory results according to study schedule
Primary and secondary endpoints according to study schedule
Comment by the investigator

8.1.4. Wash out phase

Required information:
Routine laboratory results according to study schedule
Primary and secondary endpoints according to study schedule
Comment by the investigator

8.1.5. Phase 2 (T4-T6)

Required information:
Routine laboratory results according to study schedule
Primary and secondary endpoints according to study schedule
Comment by the investigator

8.1.6. Phase 3 at T7

Required information:
Routine laboratory results according to study schedule
Primary and secondary endpoints according to study schedule
Comment by the investigator

8.1.7. Study termination

Required information:
Routine laboratory results according to study schedule
Primary and secondary endpoints according to study schedule
Comment by the investigator

8.2. List of data directly recordable on the CRF

Not applicable

9. PATIENT SELF MEASUREMENT TO BE RECORDED IN PATIENT DIARY

Not applicable

10. ASSIGNEMENT OF STUDY TREATMENT

10.1. Randomization

This study is a multicenter, randomized, open, prospective, controlled cross-over study with two different treatments.
The randomization will be performed using block randomization with site as stratification variables.

- Stratification criterion 1: Site 10 vs Site 20

The randomization list will be prepared by the biometrician of the KKS Halle. Randomization to study treatment should occur within seven days after eligibility criteria have been met. Upon confirmation of eligibility, study subjects will be randomized to one of two treatment arms in a 1:1 ratio, according to the above mentioned stratification criteria.

Study site investigators will fax the randomization form with patient details, one per patient, containing the site, patient number and treatment mode information. The investigator will have to ask for patient treatment allocation before first HD session using one of the investigational or comparator products.

The data manager will complete the randomization form and return it to the investigator who will complete the patient identification list and file it together with the treatment allocation documents.

The randomization list will be stored at KKS Halle in a fireproof cabinet and be released after a signed clean file document has been received.

10.2. TREATMENT ALLOCATION

Treatment allocation will be according to usual delivery process to the dialysis unit. After randomization the investigator is responsible for completing the randomization list and the accountability documents to record reception of investigational and comparator products.

10.3. BLINDING

Not applicable (open label study)

10.4. EMERGENCY CODE

Not applicable (open label study)

11. ADVERSE EVENTS AND SERIOUS ADVERSE EVENTS (FOR DRUGS)

NOT APPLICABLE

12. ADVERSE EVENTS AND SERIOUS ADVERSE EVENTS (FOR MD)

12.1. DEFINITIONS (ISO 14155-2011)

12.1.1. Adverse events (AE)

(Definition from ISO14155-201)
Any untoward medical occurrence, unintended disease or injury, or untoward clinical signs (including abnormal laboratory findings) in subjects, users or other persons, whether or not related to the investigational medical device.

Note 1: This definition includes events related to the investigational medical device or the comparator.
Note 2: This definition includes events related to the procedures involved.
Note 3: For users or other persons, this definition is restricted to events related to investigational medical devices.

12.1.2. Adverse Device Effect (ADE)

(Definition from ISO14155-201)
Adverse Event related to the use of an investigational medical device

Note 1: This definition includes adverse events resulting from insufficient or inadequate instructions for use, deployment, implantation, installation, or operation, or any malfunction of the investigational medical device.
Note 2: This definition includes any event resulting from use error or from intentional misuse of the investigational medical device.

12.1.3. Serious Adverse Event (SAE)

(Definition from ISO14155-201)

Adverse Event that:

- Led to death,
- Led to serious deterioration in the health of the subject, that either resulted in:
  1) A life-threatening illness or injury, or
  2) A permanent impairment of a body structure or a body function, or
  3) In-patient or prolonged hospitalization, or
  4) Medical or surgical intervention to prevent life-threatening illness or injury or permanent impairment to a body structure or a body function,
- Led to foetal distress, foetal death or a congenital abnormality or birth defect

(Definition from ISO14155-201)

Note 1: Planned hospitalization for a pre-existing condition, or a procedure required by the CIP, without serious deterioration in health, is not considered a serious adverse event,

Note 2: For the purpose of this study, the Seriousness criteria for AE reporting will be the ones described in § 12.3.5. "Seriousness" below.

12.1.4. Serious Adverse Device Effect (SADE)

(Definition from ISO14155-201)

Adverse Device Effect that has resulted in any of the consequences characteristic of a serious adverse event.

12.1.5. Unanticipated Serious Device Effect (USADE)

Serious Adverse Device Effect which by its nature, incidence, severity or outcome has not been identified in the current version of the risk analysis report.

Note: Anticipated Serious Adverse Device Effect (ASADE) is an effect which by its nature, incidence, severity or outcome has been identified in the risk analysis report.

12.1.6. AE of Special Interest

No AE of Special Interest defined for this study.

12.1.7. Device Deficiency

Inadequacy of a medical device with respect to its identity, quality, durability, reliability, safety or performance.

Note: Device deficiencies include malfunctions, use errors, and inadequate labelling.

All device deficiencies related to the identity, quality, durability, reliability, safety or performance of an investigational medical device shall be documented throughout the clinical investigation and appropriately managed by the sponsor.

Device deficiencies that did not lead to an adverse event but could have led to a medical occurrence shall be reported as a SADE to Regulatory Authorities if:

a) Either suitable action had not been taken,
b) Intervention had not been made, or
c) Circumstances had been less fortunate.

Note 1: Device Deficiencies shall be reported by the Investigator for both investigational product and potential comparator in the Device Deficiency Form provided in the CRF,

Note 2: No additional AE and SAE form have to be completed in case of DD "that could have led to a SADE".

12.2. Recording of AEs/SAEs

Adverse events, whether volunteered by the subject, discovered during general questioning by the investigator, or detected through physical examination, laboratory test or other means will be recorded on the Adverse Event sheet of the Case Report Form and followed carefully until they resolve. Abnormal laboratory values or test results should not generally be considered as adverse events, unless they induce clinical signs or symptoms or require therapeutic intervention, when they should be recorded on the Adverse Events case report form using an appropriate diagnostic description.
As far as possible, each adverse event will also be described by:

1. Its duration
2. The severity grade
3. Its relationship to the investigational or comparator product
4. The action(s) taken.

Any patient death occurring during the study shall be considered as a SAE and the cause of death form shall be attached to the “SAE Form”.

Any new medication (or unusual decrease or increase in dose for existing medication) that could induce the completion of an AE form, will be carefully checked by the Study Monitor with the Investigator.

12.3. Evaluation of AEs/SAEs

12.3.1. Intensity

The investigator will make an assessment of intensity for each AE reported during the study. The assessment will be based on the investigator’s clinical judgment. The intensity of AE recorded in the CRF should be assigned to the following categories:

- Mild: An event that is easily tolerated by the patient, causing minimal discomfort and not interfering with everyday activities,
- Moderate: An event that is sufficiently discomforting to interfere with normal everyday activities,
- Severe: An event that prevents normal everyday activities.

Comment: The term severity is often used to describe the intensity (severity) of a specific event. This is not the same as “seriousness”, which is based on patient/event outcome or action criteria.

12.3.2. Causality

The relationship (i.e. causality) between the medical device and the occurrence of each AE will be assessed and categorized as follows. The investigator will use clinical judgment to determine the relationship. Alternative causes, such as natural history of the underlying diseases, concomitant therapy, other risk factors etc., will be considered. The Investigator will also consult the reference document (i.e. Investigator Brochure, Product Leaflet).

- Not related: Temporal relationship of the onset of the event, relative to administration, implementation or misuse of the product, is not reasonable or another cause can by itself explain the occurrence of the event,
- Related:
  - Possible: Temporal relationship of the onset of the event, relative to administration, implementation or misuse of the product, is reasonable but the event could have been due to another, equally likely cause,
  - Definitely related: Temporal relationship of the onset of the event, relative to administration of the product, is reasonable and there is no other cause to explain the event, or a re-challenge (if feasible) is positive.

Note 1: All Adverse Event judged by either the investigator or the Sponsor as having a reasonable "suspected" (i.e. assessed as "Possible" or "Definitely related") causal relationship to an investigational study product qualify as Adverse Device Effect,

Note 2: Gambro Medical Assessor decision is specifically needed before any direct reporting. If follow-up information of the SAE leads to a change of assessment, a follow-up report is also submitted.

12.3.3. Occurrence

During treatment:
An event arising during the procedure of treatment that includes one of the studied products (e.g. the HD session duration, or period of use of the investigational or comparator product),
Between treatments:
An event arising in between two procedures of treatment. This includes between ICF signature and treatment or an event arising after treatment (e.g. inter dialysis period for HD, or period during which the study product is not used).

12.3.4. Expectedness

Following definition should apply to define if an Adverse Device Effect is unanticipated (i.e. unexpected) or anticipated (i.e. expected):

- Unanticipated: Adverse Device Effect should be considered as unanticipated if its nature; incidence, severity or outcome has not been identified.
- Anticipated: Adverse Device Effect should be considered as anticipated if its nature; incidence, severity or outcome has been identified.

Expected adverse events are categorised in relation to RRT:

- Complications related to central venous access
- Exit site infection
- Hypotensive episodes during RRT
- Cardiac arrhythmia
- Septicaemia
- Endocarditis
- itching
- Fluid overload
- Peripheral oedema
- Hypoalbuminemia
- Hypophosphatemia, hypomagnesemia, hypokalaemia, hypocalcaemia (all hyperdisturbances)
- Hypothermia

An unexpected adverse event is any event that is not listed as an expected adverse event in the section above.

12.3.5. Seriousness

An Adverse Event or Adverse Device Effect is considered serious if it meets one or more of the following criteria:

- Results in death,
- Is life-threatening,
- Requires inpatient hospitalization or prolongation of existing hospitalisation,
- Results in persistent or significant disability or incapacity,
- Is a congenital anomaly or birth defect.

Note:

- This definition will be the one used for the study for AE reporting,
- Life-threatening in the definition of a serious adverse event or serious adverse device effect refers to an event in which the subject was at risk of death at the time of event; it does not refer to an event which hypothetically might have caused death if it was more severe,
- Medical judgment should be exercised in deciding whether an adverse event/device effect is serious in other situations. Important adverse events/device effects that are not immediately life-threatening or do not result in death or hospitalization but may jeopardize the subject or may require intervention to prevent one of the other outcomes listed in the definition above, should also be considered serious,
- Any suspected transmission via a medicinal product of an infectious agent is also considered a serious adverse reaction.
- Planned hospitalization for a pre-existing condition, or a procedure required by the protocol, without serious deterioration in health, is not considered a serious adverse event.

12.4. Reporting of AEs / SAEs / DDs and Follow-Up

For documentation and reporting the current German regulations and local laws are applicable. The specific German documentation and reporting requirements to BfArM and associated forms are detailed explained in the *"Local Safety Reporting Process"*. Training will be performed during the initiation visit in each study site.

All safety events, serious or not, will be reported by the Investigator in the AE (and SAE forms if serious) and Device Deficiency (DD) forms. Reporting shall include all events from the time when a patient starts study specific treatment (first dialysis session of the run in phase) up to the end of the study for the concerned patient.

All AEs, SAEs and DDs observed by the Investigator or spontaneously reported by patients during the course of the study shall be recorded and described in the CRFs at each visit.

Safety events shall be reported as follows:

- All AEs, SAEs observed by the Investigator or spontaneously reported by patients during the course of the study shall be recorded in the CRF, whether considered causally related to the investigational product or not,
- All DDs shall be recorded in the CRF,
- The investigators have to inform the Study Manager
  - Of any SAE, SADE and DD occurring during the course of the study, within 24 hours from the investigator being aware of the SAE, SADE or DD, by fax, using the expedited report appended to the CRF together (for SAE and SADE) with the related AE report and required pages,
  - Of any AE assessed as "possible" or "definitively" related to the investigational or comparator product or its improper handling, within 48h after the occurrence of the event,
- The Investigator has also to notify all SAE, SADE and DD that "could have led to a SADE" to the Competent Authorities
- After internal assessment of each reportable event by the Sponsor, needed Regulatory Authorities will be notified as soon as possible, according to each country regulation, followed by as complete report as available.

Specific guideline (e.g. training material) will be given to each investigator before the first patient enrolment with details of the procedures for reporting all safety events (AE, ADE, SAE, SADE, DD) to the Sponsor, Ethics Committee and Competent Authority, in accordance with applicable local regulations. Guideline must be adapted depending on a paper or an eCRF is used for the study.

This guideline must include a definition of each type of safety events and the timing for such reporting, including the delay within the investigator must stop the reporting of new safety events that could occur after

the end of the study of the last patient (delay adaptable according to the protocol methodology, per default: 48h).

Safety events at study end for a dedicated site (i.e. Last Patient Out for the site even if the study continues in other sites) will be ensured site per site as follows (can be part of the whole DCF process management):

- DCFs will be sent to the site (for all the patients of the site) to solve all the not yet recovered AEs/SAEs, whatever their causality,
- One month later, unsolved DCFs will be sent to the site only for the AEs/SAEs/DDs related to the investigational or comparator product, its improper handling or to protocol related procedures..

12.5. SAFETY REPORT

Not applicable

13. ETHICS AND REGULATORY ISSUES

The study will be conducted in accordance with current local regulations and international applicable regulatory requirements (ISO 14155-2011, European Directive 2007/47/EC, European Directive 95/46/EC relating to data protection and the Declaration of Helsinki, last amendment in Seoul October 2008 (www.wma.net)).

13.1. ETHICS COMMITTEE

The sponsor (or investigator where applicable) is in charge of submissions to Ethics Committee. In case of investigator shall submit to EC, Gambro will provide investigator with all needed documentation/data needed for EC submission.

The Study Manager must receive a copy of the approval document supplied by the Ethics Committee, clearly identifying the protocol subjected to the committee's approval, prior to the enrollment of patients in the study and before sending investigational or comparator products and CRFs to the study sites.

During the study, the investigator shall report to the Ethics Committee any serious adverse event and/or any amendment to the protocol, in accordance with local requirements. All the correspondence with the Ethics Committee must be filed by the investigator.

Any additional requirements imposed by Ethics Committee shall be followed during the study, if applicable.

13.2. COMPETENT AUTHORITY

The sponsor is in charge of submissions to Competent Authority (CA).

The Study Manager must receive a copy of the approval document supplied by the Competent Authority, clearly identifying the protocol subjected to the CA's approval, prior to the enrollment of patients in the study and before sending investigational or comparator products and CRFs to the study sites.

During the study, any additional requirements imposed by Competent Authority shall be followed, if applicable.

13.3. AMENDMENTS

No amendment to study procedures shall be made without the mutual agreement of the Steering Committee and Gambro. All such modifications must be duly documented and signed, and are subject to protocol amendments. If any substantial changes are made to the study design, the Ethics Committee and Competent Authority shall be informed and, when necessary, shall approve these changes prior to the enrolment of new patients except in emergency case for patient's safety.

The Gambro Study Manager/Monitor is responsible for the distribution of an amendment to the Competent Authority, investigators and other persons that are involved in the study. Investigators are responsible for the distribution of this amendment to the members of their team and to the concerned Ethics Committee.

13.4. CONDITION FOR MODIFYING OR TERMINATING THE STUDY

Any change or addition to this protocol requires a written protocol amendment (which can be substantial or not according to regulation) that must be approved by Gambro and the Steering Committee before implementation. Amendments affecting the safety of subjects, the scope of the investigation or the scientific quality of the study, require additional approval of the Ethics Committee of all centers and, in some countries, of the Competent Authority. A copy of the written approval of the Ethics Committee, which becomes part of the protocol, must be given to Gambro.
Examples of amendments requiring such approval are:

- A significant change in the study design (e.g. addition or deletion of a control group),
- An increase in the number of invasive procedures to which subjects are exposed,
- Addition or deletion of a test procedure for safety monitoring.

These requirements for approval should in no way prevent any immediate action from being taken by the investigator or by Gambro in the interest of preserving the safety of all subjects included in the study. If an immediate change to the protocol is felt to be necessary by the investigator and is implemented by him/her for safety reasons, Gambro should be notified and the Ethics Committee should be informed within 10 working days.
Amendments affecting only administrative aspects of the study do not require formal protocol amendments or Ethics Committee approval, but the Ethics Committee of each centre must be kept informed of such administrative changes, e.g. changes in the staff used to monitor studies (e.g. Gambro staff versus a CRO).
Gambro may suspend or prematurely terminate either the clinical study in an individual investigation site or the entire clinical study for significant and documented reasons.

13.5. PATIENT DATA PROTECTION

Each patient must be identified on the CRF with his identification number indicating his rank of inclusion into the study. Investigators must keep the confidential patient identification list of all the patients, including identification numbers, full names, date of birth and last known addresses (patient identification list).
Patients must be informed in writing about the possibility of audits by authorized representatives of the company and/or regulatory authorities, in which case the relevant parts of study-related hospital records may be required.
Patients must also be informed that the results obtained will be computer-stored and analyzed, that study data may be shared with or transferred to Gambro's affiliates outside of country where they signed their consent form without further notice to the patient, that local laws must be applied, that patient’s confidentiality and personal information must be preserved and remain confidential, and that they are entitled to obtain any information concerning the data stored and analyzed by a computerized system.

13.6. PATIENT’S INFORMATION AND INFORMED CONSENT

The investigators must ensure that the patients have received all relevant information, orally or in writing, relating to the type, objective and possible risks and benefits of the study. Patients must also be informed that they are free to withdraw from the study at any time.

The information will be given in reasonable time before study start.

The investigators must obtain written informed consent from the patients prior to their enrolment in the study. The information given to the patient must be attached to the written informed consent.

If modifications are needed according to local regulations, or if new information becomes available (e.g. from Sponsor) that can significantly affect a subject's future health and medical care, a new version of the “Patient information and informed consent” must be prepared in cooperation with investigator(s) and approved by

Gambro. The new version of the “Patient information and informed consent” shall be given to each enrolled patient for information and agreement.

13.6.1. General process for obtaining informed consent

The general process for obtaining informed consent will be followed by Sponsor and Investigator and shall:

a) Ensure that the principal investigator or his/her authorized designee conducts the informed consent process,
b) Include all aspects of the clinical investigation that are relevant to the subject's decision to participate throughout the clinical investigation,
c) Avoid any coercion or undue improper influence on, or inducement of, the subject to participate,
d) Not waive or appear to waive the subject's legal rights,
e) Use native non-technical language that is understandable to the subject,
f) Provide ample time for the subject to read and understand the informed consent form and to consider participation in the clinical investigation,
g) Include personally dated signatures of the subject and the principal investigator or an authorized designee responsible for conducting the informed consent process,
h) Provide the subject with a copy of the signed and dated informed consent form and any other written information,
i) Show how informed consent will be obtained and recorded in special circumstances where the subject is unable to provide it him- or herself, and
j) Ensure important new information is provided to new and existing subjects throughout the clinical investigation.

14. ARCHIVING OF STUDY DOCUMENTATION

The investigators must keep essential study documents (including all original raw data together with the patients’ identification list and signed informed consents) minimum 5 years after product commercialization is stopped and should be retained for a longer period however if required by the applicable regulatory requirements or by an agreement with the sponsor.
Patient medical files have to be kept as much as possible at each investigation site.
Investigators will be informed by Sponsor when documents related to the study could be deleted. Prior to destroying study-related documentation, the investigator shall make sure with the Sponsor, which it does not need to be kept any longer.

15. INVESTIGATIONAL PROCEDURES

15.1. TRAINING

The Gambro Study Monitor(s) will ensure that hospital staff involved in the study (namely Principal Investigator team, Pharmacy, Laboratory) receives appropriate training for the purpose of the study.

Both Gambro and the investigators must make sure that sub-investigators and any other staff participating actively in the study have been appropriately trained and received the relevant information relating to the performance of this study.

If needed, a second training can be organized on study site request for clarification purposes.

All trainings shall be recorded in a Training Log and file in the Study Master File and respective Master files (e.g. Investigator Site File).

15.2. MONITORING

During the course of the study the Gambro monitor(s) will have regular contacts with the study sites and will make regular visits to get an overseeing of the clinical study. Before the patient enrolment starts and during the course of the study, the Gambro monitor(s) will ensure that clinical facilities are acceptable, that the accountability of the investigational and comparator product is performed, that the clinical study is conducted, recorded, and reported in accordance with the protocol, written procedures (see CTF BG 003 Study Monitoring Process), the current regulation linked to the investigational or comparator product type (see § Ethics and Regulatory Issues), and the applicable local regulatory requirements. The Gambro monitor(s) will also ensure that source data are available and that the study results are properly recorded in CRFs.

By signing the protocol, the investigators agree to allocate their time and the time of their staff to discuss findings and any relevant issues during the monitoring visits.

The study monitor must fill in a monitoring report at each study visit according to the monitoring procedure. The monitoring report will include namely information on the following:

- Patient’s informed consent obtained prior to the start of the study,
- Patient’s status (enrolled, included, withdrawn and completed),
- Verification of source data according to the monitoring check-list,
- Compliance with inclusion and exclusion criteria according to the monitoring check-list,
- Compliance with sampling procedure,
- Potential deviation from protocol (namely if the deviation affects subject's rights, safety and wellbeing, or the scientific integrity of the clinical investigation),
- Safety issues (i.e. potential observed AE, SAE, Device Deficiencies and Other Observations).

15.2.1. Deviations from clinical investigation plan

The investigator is not allowed to deviate from the protocol. Under emergency circumstances, deviations from the protocol to protect the rights, safety and well-being of human subjects may proceed without prior approval of the sponsor and the EC. Such deviations shall be documented and reported to the sponsor as soon as possible.

- The Study Monitors specify in the Monitoring Visit all deviation from protocol (namely if the deviation affects subject's rights, safety and wellbeing, or the scientific integrity of the clinical investigation),
- The Study Manager checks the pertinence of the deviation,
-
- If a deviation is effective, the Investigator shall be informed (formal letter) to prevent potential future deviation, and the deviation is listed in a table (see CTF BG 004 “Data Management”),
-
- The Deviation table shall be taken into account during the final data review (Adherence Review Meeting).

15.3. DIRECT ACCESS TO SOURCE DOCUMENTS

The investigator must give the Study Monitor direct access to relevant hospital or clinical records to confirm their consistency with the CRF entries. No information in these records about the identity of the subjects will leave the study site. Gambro monitoring standards require full verification for the presence of informed consent, adherence to the inclusion/exclusion criteria, documentation of AEs/SAEs and Device Deficiencies and the recording of primary efficacy and safety variables. Additional checks of the consistency of the source data with the CRFs are performed according to the study-specific monitoring plan.

15.4. Source data verification

By signing the protocol, the investigators agree to allocate their time and the time of their staff to discuss findings and any relevant issues linked to source data.
A study-specific monitoring plan specifies which and whether other parameters need to be verified by 100%
The data to be compulsorily checked are the following:

- Inclusion and exclusion criteria,
- Adverse Events, Serious Adverse Events and Device Deficiencies (for MD),
- Concomitant medication (when possible effect on evaluated parameters),
- Main criteria laboratory data.

Note: Study Manager can decide to adapt the % of verification of parameters during the study.

15.5. Audits and inspections

In addition to the routine monitoring procedures and in accordance with Good Clinical Practice (GCP) principles, GCP audits might well be performed by the Quality Assurance members of Gambro. These audits of clinical research activities are in accordance with applicable regulatory requirements, Gambro internal policies and procedures, to evaluate compliance with the principles of GCP. A Regulatory Authority may also wish to conduct an inspection (during the study or even after its completion). If a Regulatory Authority requests an inspection, the investigator must immediately inform the Study Monitor of Gambro that this request has been made.

16. STUDY ORGANIZATION

16.1. Investigators

The investigators taking part in the clinical study are nephrologists with wide experience in the field of renal replacement therapies including HD treatments. They already have experience in conducting clinical studies according GCP.

Sponsor shall receive disclosures of conflict of interest from coordinating investigators, principal investigators and sub-investigators, <u>where required by national regulations</u>.

16.1.1. Coordinating and Principal Investigators

See page 1-3 or “Contact List”

16.2. Study administration

Gambro has established a study team comprising a Study Manager and Study Monitor (see Contact List). This study team missions are to ensure the compliance with protocol by following GCP as well as planning follow-up.

16.2.1. Steering Committee

The Steering Committee is the main policy and decision making committee of the study and has final responsibility for the scientific conduct. The Steering Committee is free to choose and invite any expert they regard as necessary. Contact details of the Steering Committee are presented in the “Contact List”.

16.2.2. Data Safety Monitoring Board (DSMB)

Not applicable

16.3. Other Study contacts

16.3.1. CRO

Contact details of the CRO KKS Halle are presented in the “Contact List”

17. STATISTICAL METHODS

17.1. Population size estimation

.

17.1.1. Sample size calculation

The sample size estimation is based on results of the Perci 1 Study. In this study we found an effect size (intraindividual difference of means divided by intraindividual standard deviation) of about 0.5 for the TNF alpha expression. Assuming a power of 80%, a level of significance of 0.05 (two-sided) we need 34 evaluable patients. Taking into account a 30% drop out rate we have to allocate 50 patients to obtain 35 evaluable patients. This number is based on an observed drop-out rate of 20% in the Perci 1 study with shorter study duration. Note that the primary analysis will be done baseline adjusted and we expect an increase of power due to this adjustment. Further, we expect that the decrease of variance due to stratification and baseline adjustment will more than outweigh the loss of degrees of freedom due to adjustment and stratification.

17.2. Coding

All AEs will be categorized by the Medical Dictionary for Regulatory Activities (MedDRA).

17.3. Statistical analysis

A complete description of the statistical analysis will be presented in the Statistical Analysis Plan which should be final, approved and signed before database clean file.

17.4. Statistical analysis

A complete description of the statistical analysis will be presented in the Statistical Analysis Plan which should be final, approved and signed before database clean file.

Primary analysis:
The primary analysis compares the expression values of TNF alpha intraindividually between both membranes. The null hypothesis and alternative are

H0: There is no difference between both membranes regarding the expression of TNF alpha
H1: There is a difference between both membranes regarding the expression of TNF alpha

Even so the alternative is formulated undirectedly and the significance testing will be carried out two-sided we expect superiority of HCO vs the control membrane, i.e. lower expression rates for the HCO membrane. The analysis will be done by testing the main effect of treatment in a GEE (Generalized Estimating Equations) model with baseline adjustment, including effects for time and centre as additional covariates. The level of significance is 0.05 (two-sided).

Study populations

In a blind data review (see below) each patient will be assigned to a study population

PP includes all patients treated according to the study protocol including those with minor deviations to be defined before the blind data review

(Modified) ITT includes additionally to PP all patients with measurements from at least one study phase and no clinical events which make measurement of primary endpoint impossible

Full Set includes all randomized patients

Safety includes all patients which received at least one intervention

Primary analysis population:
The primary analysis population will be the modified ITT population. Patients with clinical events making the analysis of the primary endpoint impossible will be excluded after a blind data review. The remaining drop outs will be included in the analysis if results are available for at least one treatment phase.

Secondary analyses
The secondary analyses include the analysis of the primary endpoint in the per protocol population. Secondary outcome variables will be analysed in the same way as the primary endpoint, using generalized linear models and the method of GEE as described above.

Descriptive analyses
The description includes means, medians, standard deviations, quartiles and ranges according to scaling and distribution of the variables. Graphical descriptions include line graphs displaying the individual measurement over time for each patient and box plots.

Exploratory analyses
1. In exploratory analyses we will investigate which measurements at baseline might be prognostic for the performance of the several membranes. This analysis will be, however, of limited power due to the moderate sample size.
2. The analysis of Phase 3 of the trial (prolongation of treatment after the termination of the second period of the cross over design) will be exploratory. Analysis will be done using generalized estimating equations including a three level factor for time and the two level factor for treatment. The primary hypothesis within this analysis is the treatment effect. Baseline will be adjusted for analogously to the primary analysis. Time vs treatment interaction will be investigated.

Interim analyses
No interim analyses are planned.

Subgroup analyses
The different study centers will be analyzed separately. This, however, is an exploratory analysis and there might be not enough power to confirm superiority of the HCO membrane within centers.

Blind data review
For the blind data review the data management will provide sufficient data to the statistical centre which allow to assign subjects to the study populations described above but blinded for treatment arm and with primary endpoint data deleted. If necessary, variables different from study arm which allow a probable guess for the study arm will be excluded. The statistical centre will provide a listing with relevant information and a proposal for assignment of population to the principal investigators of both study sites, presenting to each PI data from the other study site only to obtain a maximum degree of blinding. The blind review meeting will be face to face or as a telephone conference.
.

18. DATA MANAGEMENT

18.1. Case report form

The Data management team is responsible for the creation of the paper-based study CRF

CRF completion rules are provided to the Investigator during the initiation visit via the "CRF Completion Guideline".

One CRF per patient must be completed for all patients enrolled in the study. All the boxes provided in the CRF must be filled in. Investigators' or his delegated assistants' signatures must attest the accuracy of data entered in all CRFs.

Data editing for correction or clarification purposes (i.e. Data Clarification Forms, DCF) must be done before the case report forms have been transmitted for final data processing and analysis. Subsequent corrections must be checked and validated in signing by the investigators or his delegated assistants. Originals of completed CRFs are the property of Gambro.
The investigators must ensure that CRFs are accessible to the Study Monitor, the Study Manager or any authorized people at any time during the course of the study.

Paper CRFs must be filled on white page in using a permanent black ballpoint pen and corrections can only be made by crossing out incorrect data and entering the correct data close to those crossed out. Data must not be erased in any manner whatsoever. Modifications made by the investigator or his delegated assistants must be signed and dated. The Study Monitor, in collaboration with the investigators, must ensure that data entered in the CRFs are correct and legible.

18.2. Data entry and storage

The entry of CRF data into the CRF is the responsibility of the investigator. The transfer of the CRF from the study site to KKS Halle is the responsibility of the sponsor.
The study management software eResearchNetwork, a commercially available validated software approved by the Food and Drug Administration (FDA), will be used for data capture and query management. This database further allows the audit trail of any modifications made during data entry.
Data entry will be carried out by a single person and validated by independent "second look".
Data storage will be done according to the national regulations.

18.3. Data management and quality control

Database setup, validation, data management and database lock will be performed by the KKS Halle according to the appropriate SOPs.

All data management tasks and responsibilities will be described in the study Data Management Plan (DMP), as part of the study master file. Data consistency will be checked according to the procedure described in the Data Management Plan (DMP) and in the Data Validation Plan (DVP).

The DMP is approved by the study manager T. Böhler (Gambro), the study data manager Y. Ronniger (KKS Halle) and the statistician (P. Martus).

Data will be evaluated for consistency, accuracy and completeness regularly. After the database has been declared complete and accurate, the database will be locked. Any changes to the database after that time can only be made by joint written agreement between the Coordination Investigator and the trial statistician. SAS data files will be transferred to the trial statistician for the final analyses.

19. ADMINISTRATIVE PROCEDURES

19.1. STUDY DOCUMENTS AND RECORD KEEPING

The investigator is responsible for completing the CRFs and the Study Monitor is responsible for reviewing them and clarifying with the investigator any data queries, if any. The completed and corrected CRFs for completed visits will be collected by the Study Monitor initially, and sent for data processing. A copy of the CRFs is retained by the investigator who must ensure that it is stored with other study documents, such as the protocol, the investigator's brochure and any protocol amendments, in a secure place, with restricted access. Data on subjects collected on CRFs during the study will be documented in an anonymous fashion and the subject will only be identified by the subject number. If, as an exception, it is necessary for safety or regulatory reasons to identify the subject, the Study Monitor and the investigator are bound to keep this information confidential.
The investigator must maintain source documents for each subject in the study. All information on CRFs must be traceable to these source documents, which are generally maintained in the subject's medical file.

For each patient, source documents must clearly and at least specify the following:

- Participation of the patient in the study (patient and study's identification),
- A copy of the signed informed consent which must indicate the study number and title of the study,
- All demographic and medical information, including laboratory data, electrocardiograms, etc., concomitant treatments or medications,
- Any visit to the hospital, particularly those visits made for the sole purposes of the study,
- Safety events (AEs, SAEs, Devices Deficiencies),
- Potential deviations to the protocol.

Study Essential Documents must be retained by the investigator for as long as needed to comply with national and international regulations. Essential documents include all those mentioned under ICH GCP E6 Guideline Section 8. The investigator agrees to adhere to the document-retention procedures by signing the protocol.

19.2. CONFIDENTIALITY AND DATA PROTECTION

By signing the protocol, the investigator agrees to keep all information provided by Gambro in strict confidentiality and to request similar confidentiality from its staff. Study documents provided by Gambro (protocols, investigators' brochure, CRFs and other material) will be stored appropriately to ensure their confidentiality. The information provided by Gambro to the investigator may not be disclosed to others without direct written authorization from Gambro, except to the extent necessary to obtain informed consent from subjects who wish to participate in the study.

19.3. FINANCING AND INSURANCE

19.3.1. Fixed duty

Gambro Company, sponsor of this clinical study, will pay the fixed duty of fees to the local Ethical Committees and application fees to the competent authorities.

19.3.2. Insurance

Concerning the direct or indirect responsibility of the investigational product during this clinical study, Gambro is liable, on behalf of investigator and his/her assistants, for any damage caused to the patient, provided that the investigators and assistants have complied with Gambro's instructions specified in this protocol and related amendments, that the investigational product used during this clinical study has been supplied by Gambro, and that the investigators and their assistants have conducted this clinical study in accordance with the Good Clinical Practice (GCP) requirements, scientific practice, techniques and know-how in force.

Gambro Company's liability is covered by an insurance policy signed to *ACE European Group Limited* (insurance policy no. 43GEA10524) as required by national regulations.

19.4. RESULT PROPERTY

19.4.1. Confidentiality

Given the requirements of this clinical study, confidential, clinical and patient-related information can be brought to the knowledge of the Study Manager appointed for this study. Gambro requires that the Study Manager preserves the confidentiality of such information, which should under no circumstance be disclosed or communicated to third parties outside Gambro company (except CRO) or any other member of Gambro personnel not involved in the study. CRFs may be communicated to the authorized representatives of the relevant regulatory authorities and people in charge of the evaluation of the applications for registration on behalf of Gambro.

19.5. PUBLICATION OF STUDY RESULTS

As well conducted research is the basis for treatment decisions and improvement of public health, results of this clinical study are intended, whatever they are, to be published (i.e. current protocol will be prospectively registered in European or US database before first patient enrolment and result will be further registered).

The results of this clinical study will be presented to the investigator when the analysis has been completed. On the grounds of these data, the sponsor, in collaboration with the investigator, will write a final study report. The study report may also be submitted to a respected international peer-reviewed journal. They may also be submitted as abstracts to a scientific congress for oral or poster communication, the authors, schedule and provisions having been previously defined by the investigator and the sponsor. No study data shall be published, presented or communicated to third parties, except for regulatory data, prior to the authorized date. Both the investigator and the sponsor have at least 30 days to revise or comment any manuscript, poster, etc., prior to submission of the results for publication or presentation purposes.
As this is a multicentric study no single center publication is allowed on the core data unless for safety concern. Ancillary studies performed in one or more centers may be published in separate papers. It is the responsibility of the Steering Committee to interpret data globally and no publication will be made without allowing the Steering Committee and the sponsor to review the paper and make comments that should be reported in the publication.

20. REFERENCES

1 ISO 14155-2011(E) “Clinical investigation of medical devices for human subjects – Good clinical practice.

2 ICH “Harmonized Tripartite Guidelines”: Guideline for Good Clinical Practice E6(R1), Current Step 4 Version, dated 10 June 1996.

3 Declaration of Helsinki, last amended in Seoul, October 2008 (www.wma.net),

4 Directive 2007/47/EC of the European Parliament and of the Council of 5 September 2007,

5 Directive 1995/46/EC of the European Parliament and of the Council of 24 October 1995,

6 Directive 1993/42/EC of the European Parliament and of the Council of 14 June 1993,

21. LIST OF APPENDICES

Not applicable
