# Supplementary material for: Medium Cut-Off (MCO) Membranes Reduce Inflammation in Chronic Dialysis Patients—A Randomized Controlled Clinical Trial
Source: PLoS One. 2017 Jan 13;12(1):e0169024. doi: 10.1371/journal.pone.0169024 (PMC5234772; doi:10.1371/journal.pone.0169024)
Supplement: S3 File — (PDF) [file pone.0169024.s003.pdf]

Supplementary statistic data

In the following definitions the abbreviation CRO refers to the crossover part (including two periods) and the abbreviation EXT to the extension part.

FS Population (Full Set): Includes all randomized patients

PP-CRO Population (Per Protocol): Includes all patients treated according to the study protocol during the crossover part including those with minor deviations as defined before the blind data review

(Modified) ITT-CRO Population (Intent To Treat): Additionally to PP-CRO includes all patients with measurements from at least one period of the crossover part and without clinical events which make measurement of primary endpoint impossible.

PP-EXT Population (Per Protocol): Includes all patients treated according to the study protocol during the entire study (both periods of the crossover part and the extension part), including those with minor deviations (as defined before the blind data review).

(Modified) ITT-EXT Population (Intent To Treat): Includes additionally to PP-EXT all patients which are PP-CRO or ITT-CRO and which contribute measurements from at least one visit of the extension part and which had no clinical events which make measurement of the primary endpoint impossible during the extension part.

SAF Population (Safety): Includes all patients which received at least one intervention
